# Synthesis and Dynamic Behavior of Ce(IV) Double-Decker Complexes of Sterically Hindered Phthalocyanines

**DOI:** 10.3390/molecules29040888

**Published:** 2024-02-17

**Authors:** Jeevithra Dewi Subramaniam, Toshio Nishino, Kazuma Yasuhara, Gwénaël Rapenne

**Affiliations:** 1 Division of Materials Science, Nara Institute of Science and Technology, 8916-5 Takayama, Ikoma 630-0192, Japan; 2 Center for Digital Green-Innovation, Nara Institute of Science and Technology, 8916-5 Takayama, Ikoma 630-0192, Japan; 3 Centre d’Elaboration de Matériaux et d’Etudes Structurale, Université de Toulouse, CNRS, 29, Rue Marvig, 31055 Toulouse, France

**Keywords:** double-decker complex, homoleptic, heteroleptic, cerium ion, ligand rotation, phthalocyanine, carbazole, phenothiazine

## Abstract

Phthalocyanines and their double-decker complexes are interesting in designing rotative molecular machines, which are crucial for the development of molecular motors and gears. This study explores the design and synthesis of three bulky phthalocyanine ligands functionalized at the α-positions with phenothiazine or carbazole fragments, aiming to investigate dynamic rotational motions in these sterically hindered molecular complexes. Homoleptic and heteroleptic double-decker complexes were synthesized through the complexation of these ligands with Ce(IV). Notably, **Ce^IV^(Pc2)_2_** and **Ce^IV^(Pc3)_2_**, both homoleptic complexes, exhibited blocked rotational motions even at high temperatures. The heteroleptic **Ce^IV^(Pc)(Pc3)** complex, designed to lower symmetry, demonstrated switchable rotation along the pseudo-C_4_ symmetry axis upon heating the solution. Variable-temperature ^1^H-NMR studies revealed distinct dynamic behaviors in these complexes. This study provides insights into the rotational dynamics of sterically hindered double-decker complexes, paving the way for their use in the field of rotative molecular machines.

## 1. Introduction

Phthalocyanines (Pc) [1] represent a class of porphyrinoids that possess intriguing electronic, optical, and magnetic properties [2]. Thanks to their unique structural features and versatile properties, they are widely used as a building block in various functional materials, such as molecular electronics [3], dyes [4], photovoltaic devices [5], and advanced catalysts [6]. Phthalocyanines can coordinate to various metal ions in their central cavity of a macrocyclic structure composed of four isoindoles bridged by nitrogen atoms. Their coordination ability is not limited to alkaline metals, alkaline earth metals, and transition metals, but also to rare-earth ions. In particular, phthalocyanines form double-decker complexes with metal ions with large ionic radii like Cd, Hg, and rare-earth ions, in which the metal ion is sandwiched with two phthalocyanines [7,8]. These double-decker complexes exhibit unique molecular properties such as multistep redox properties [9,10,11], single molecular magnetism [11,12], and photocatalytic properties [13] which are derived from metal–π and π-π interactions. Moreover, the three-dimensional architecture of these double-decker structures makes them versatile building blocks for designing molecular machines with rotary units capable of controlled motion at the molecular level such as molecular motors and gears [14,15,16,17,18,19].

The first demonstration of rotation behavior in solution involving phthalocyanine macrocycles was reported in 2011 by Otsuki et al. [20]. They studied a two-fold symmetric heteroleptic double-decker complex with a meso-substituted porphyrin and a Pc ligand coordinated to a Ce(IV) ion. The inter-ring rotation was observed as a flip by 90° from one antiprismatic geometry to another as evidenced by variable-temperature NMR. In 2023, Martynov et al. published the intramolecular rotation of Y(III) phthalocyaninates by analyzing the change in the conformational behavior, also using variable-temperature NMR [21]. However, the effect of the bulkiness of peripheral substituents on the rotative motion around the central metal ion of double-decker complexes was not well investigated for bis(phthalocyanato) double-decker complexes since the regio-controlled functionalization and desymmetrization of phthalocyanines are more difficult compared with porphyrins. This work specifically focuses on the formation of double-decker complexes using bulky Pc [20,22,23,24,25,26]. The aim is to investigate the dynamic rotation motions under thermal influence in these sterically hindered molecular complexes.

Here, we report the design and synthesis of three bulky Pc ligands functionalized at the α-positions with four *tert*-butyl phenothiazine (**H_2_Pc1**) or four *tert*-butyl carbazole (**H_2_Pc2**). Furthermore, a desymmetrized A_3_B phthalocyanine (**H_2_Pc3**) was also prepared with one phenothiazine and three *tert*-butyl carbazole substituents. These ligands were used to synthesize two homoleptic and one heteroleptic double-decker complexes through the complexation of these Pc with Ce^IV^. The internal rotating motions of these complexes were studied in solution using variable-temperature ^1^H-NMR (VT-^1^H-NMR).

## 2. Results and Discussion

### 2.1. Molecular Design of Sterically Hindered Double-Decker Complexes

**Figure 1:**
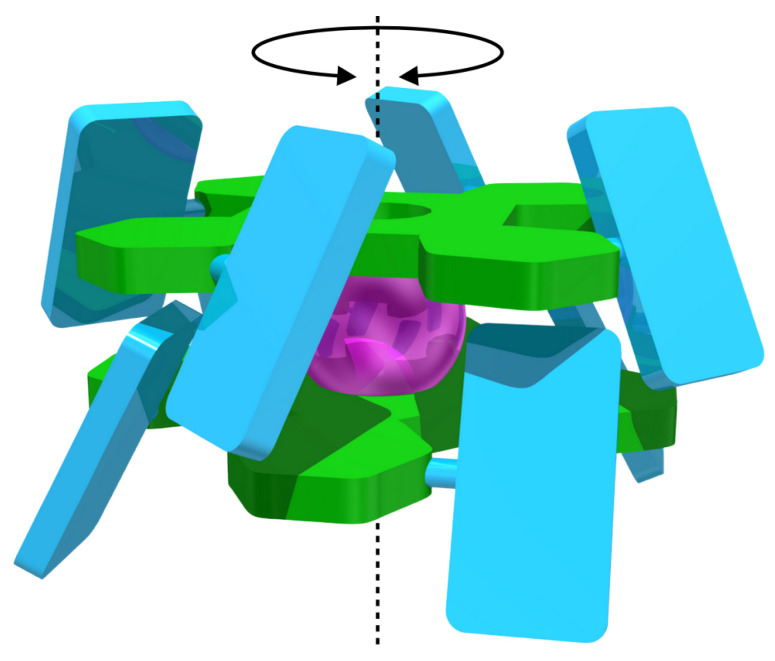
Schematic design of the sterically hindered double-decker complex of this work with the axis of rotation of interest (the main axis of rotation is given as a dashed line). The phthalocyanine rings are shown in green, the lanthanoid ion in purple, and the planar bulky substituents in blue.

Double-decker complexes with sterically hindered peripheral substituents, wherein a lanthanoid ion is sandwiched between two Pc ligands (Figure 1) can serve as valuable structural motifs for the study of intramolecular rotating motions since the ligands can rotate around the metal center (axis of rotation shown in Figure 1). To examine the impact of bulkiness on the formation of double-decker complexes and the dynamics of their rotating motions, we designed Pc ligands functionalized at the α-positions with the planar bulky substituents 3,6-di-*tert*-butyl phenothiazine and 3,6-di-*tert*-butyl carbazole. The *tert*-butyl groups are present here to improve the solubility of the target ligands and double-decker complexes. 

### 2.2. Synthesis of the Pc Ligands Functionalized at the α-Positions with Bulky Groups

**Figure 2:**
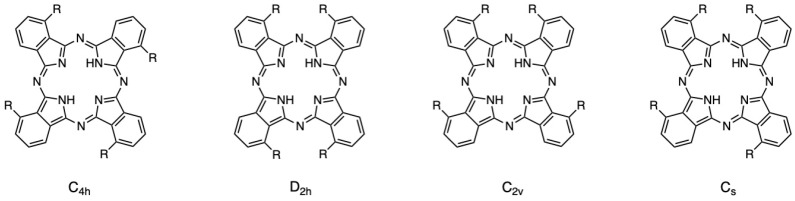
Regioisomers of the Pc ring obtained by cyclic tetramerization of 3-substituted phthalonitrile.

The cyclic tetramerization of mono-substituted phthalonitrile can produce a mixture of four distinct regioisomers with C_4h_, C_s_, C_2v_, and D_2h_ symmetries, as illustrated in Figure 2. It has been firmly established that introducing bulky substituents at the 3-position of phthalonitrile selectively yields the C_4h_ isomer [22]. The reaction progresses through heating the phthalonitrile in a solution of lithium octanolate in n-octanol [27].

**Figure 3:**
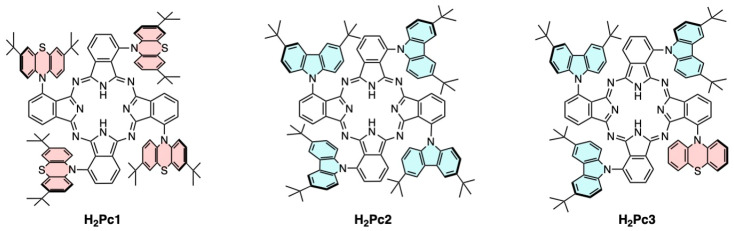
The three Pc ligands synthesized by tetramerization of phathalonitrile precursors.

Two symmetric A4 phthalocyanine ligands with four 3,7-di-*tert*-butyl phenothiazines (**H_2_Pc1**) or four 3,6-di-*tert*-butyl carbazoles (**H_2_Pc2**) at the α-position were synthesized. Moreover, a desymmetrized A3B phthalocyanine (**H_2_Pc3**) was also prepared with one phenothiazine and three 3,6-di-*tert*-butyl carbazole substituents (Figure 3). 

#### 2.2.1. Symmetric A4 Pc Ligands **H_2_Pc1** and **H_2_Pc2**

The synthesis of the phenothiazine-substituted phthalonitrile was achieved in two steps. Firstly, 3,6-di-*tert*-butyl phenothiazine was synthesized in 79% yield via a double Friedel–Crafts alkylation of phenothiazine with AlCl_3_ [28] followed by the N-arylation of the phenothiazine by 3-fluorophthalonitrile in the presence of NaH [29]. The 3,6-di-*tert*-butyl carbazolylphthalonitrile was obtained by following a modified published procedure [19]. Tetramerization of the phthalonitriles selectively gave the C_4h_ symmetric Li_2_Pc ligands through Li template synthesis. The metal-free **H_2_Pc1** and **H_2_Pc2** were then quantitatively obtained by demetallation of the lithium ions by reaction with concentrated HCl. The formation of the Pc ligands functionalized at the α-positions was confirmed by ^1^H-NMR and MS (Figures S1–S3). In particular, the ^1^H-NMR spectra revealed that, as expected, only one isomer was selectively obtained. Generally, Pc ligands tend to easily aggregate in solution, resulting in broad signals. Surprisingly, for these bulky ligands, the signals are sharp, indicating the difficulty for the aromatic rings to interact closely.

#### 2.2.2. Disymmetric A3B Pc Ligand **H_2_Pc3**

To facilitate the tracking of the rotation, we envisioned the preparation of a disymmetric A3B Pc ligand [30]. For this purpose, alongside three 3,6-di-tert-butyl carbazole subunits, we selected the phenothiazine fragment as the chemical tag to complete the molecular structure. 

**Scheme 1:**
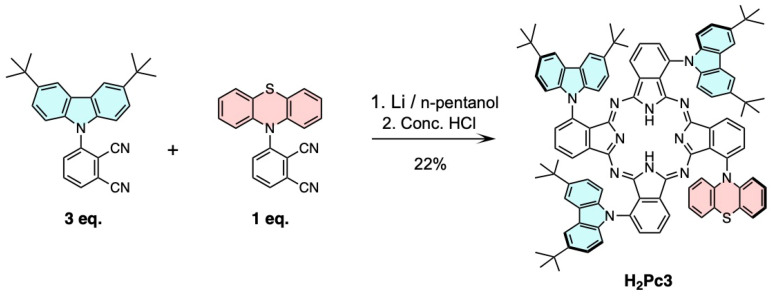
Synthesis of the disymmetric **H_2_Pc3** ligand based on a statistical condensation reaction.

The desymmetrized **H_2_Pc3** ligand was synthesized via a statistical condensation reaction. Six different compounds could be obtained, A4, A3B, A2B2 (cis and trans isomers), AB3, and B4, in a statistical distribution. Since the two dinitrile compounds are expected to exhibit comparable reactivity, employing a strict 3:1 molar ratio of each dinitrile favors the formation of the desired A3B compound with a statistical yield of 44% followed by the symmetric A4 compound (33%) and the remaining cis and trans A2B2, AB3, and B4 as minor products [31]. **H_2_Pc3** was obtained by heating a mixture of 3,6-di-*tert*-butyl-carbazole phthalonitrile with 3-phenothiazine phthalonitrile in a 3:1 molar ratio in lithium pentoxide in *n*-pentanol at reflux for 21 h (Scheme 1). Purification of the mixture by silica column chromatography yielded the desired compound in 22% yield.

### 2.3. Synthesis of the Double-Decker Cerium(IV) Complexes

**Figure 4:**
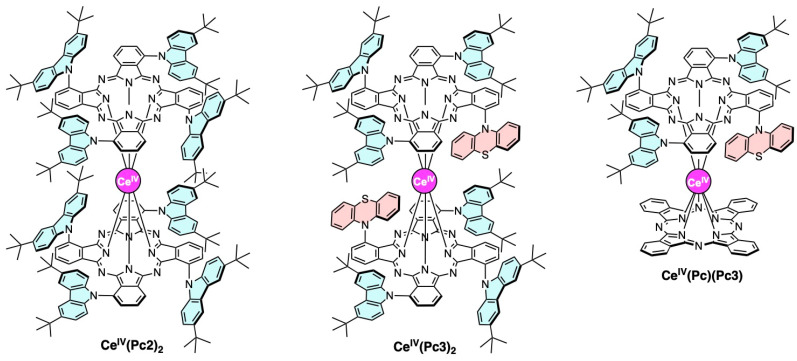
The three double-decker complexes synthesized by coordination with a cerium(IV) ion.

Figure 4 presents the three double-deckers synthesized in this work. **Ce^IV^(Pc2)_2_** and **Ce^IV^(Pc3)_2_** are homoleptic while **Ce^IV^(Pc)(Pc3)** is heteroleptic.

#### 2.3.1. Homoleptic Double Deckers **Ce^IV^(Pc2)_2_** and **Ce^IV^(Pc3)_2_**

Firstly, we attempted the synthesis of the homoleptic double-deckers with the three bulky Pc ligands shown Figure 3, using the microwave conditions described by H.G. Jin et al. [32]. 

The reaction of **H_2_Pc1** with Ce(acac)_3_·nH_2_O was initially attempted using microwave heating in three cycles of 1 h at 270 °C, but only the starting material was quantitatively recovered, as confirmed by ^1^H-NMR data. A second attempt by refluxing **H_2_Pc1** with the same cerium source in n-octanol for 8 h also failed, possibly due to excessive steric hindrance between the phenothiazine groups of two different ligands. To validate this hypothesis, we attempted to form the double-decker complex with the less hindered **H_2_Pc2**. Connected through a nitrogen atom involved in a five-membered cycle (instead of a central six-membered ring for the phenothiazine fragment), the carbazole sub-unit is less sterically hindered. Under the same reaction conditions using microwave heating, the less hindered **H_2_Pc2** successfully coordinated to the Ce(IV). The reaction was quenched with MeOH, and the precipitate obtained was purified by silica column chromatography and recycling GPC, followed by recrystallization to yield the double-decker complex **Ce^IV^(Pc2)_2_** in 70% yield.

**Figure 5:**
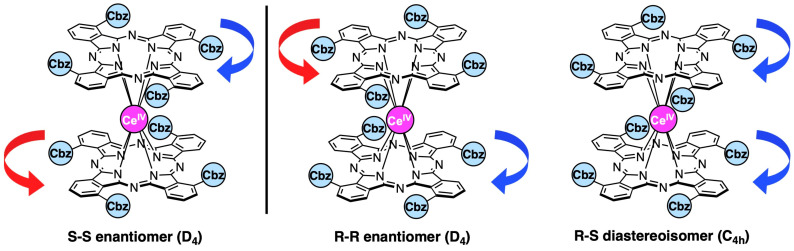
Possible stereoisomers of double-decker complex formed from a Pc functionalized at the four α-positions: R,R and S,S enantiomers as well as the R,S meso diastereomer (Cbz = 3,6-di-*tert*-butyl-carbazole).

In addition to giving neutral double-decker complexes, cerium (IV) is also one of the lanthanide ions that exhibit diamagnetism in double-decker structures [7]. Pc functionalized at the four α-positions is prochiral. Once coordinated in a double-decker architecture, as depicted in Figure 5, three stereoisomers can be envisioned [33]: one pair of enantiomers (R-R and S-S) and a meso (R-S). It is expected that the (R-S) meso form would be obtained as the main product since the steric hindrance is reduced between the upper and lower substituents [34].

**Figure 6:**
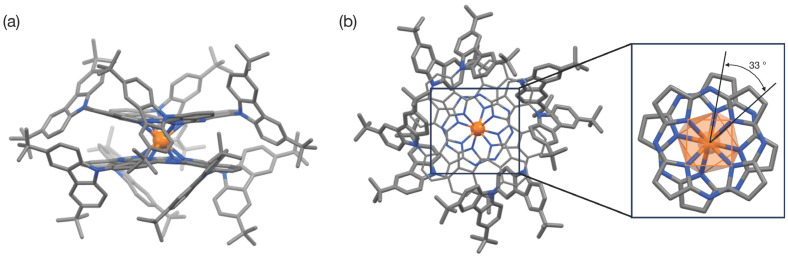
Side view (**a**) and top view (**b**) of the single crystal structure of **Ce^IV^(Pc2)_2_**. Hydrogens are omitted for clarity. Ce is in orange, N in blue and C in grey.

Single crystals suitable for X-ray diffraction analysis were obtained by a slow diffusion of methanol into a chloroform solution of the complex. Their analysis confirms the formation of the expected (R-S) meso stereoisomer of the sterically crowded double-decker complex **Ce^IV^(Pc2)_2_**. The cerium center is coordinated by eight nitrogen atoms of the **Pc2**, forming a distorted square antiprismatic coordination geometry with a twisting angle of 33° (Figure 6). 

The sterically hindered ligand drastically modifies this angle, which is usually about 45° in homoleptic double-decker complexes of Pc [19,33]. Similar to the structures of many double-decker complexes, the two ligands are not strictly planar and display a saucer shape. This structure reveals that peripheral carbazoles are arranged in a herringbone manner compared to each other. The carbazole substituents of both Pc rings are not perpendicular to the average plane of the Pc rings but are tilted at +52° in one Pc ring and −48° in the other one. This herringbone arrangement of carbazoles in the crystal could be due to the T-shaped π-π interaction between neighboring carbazoles.

**Figure 7:**
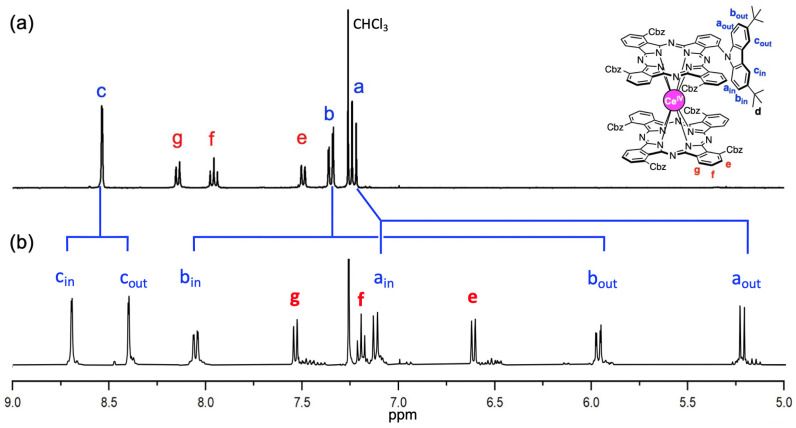
^1^H-NMR spectra of (**a**) **H_2_Pc2** and (**b**) **Ce^IV^(Pc2)_2_** in CDCl_3_ (400 MHz). The full assignments of the signals (indicated with letters in the molecule shown top right) were made with the assistance of COSY (Figure S9).

From the ^1^H-NMR spectrum of **Ce^IV^(Pc2)_2_** depicted in Figure 7, it is clear that a major isomer is present, with less intense signals corresponding to minor stereoisomers (R-R and S-S). The MS data (Figure S11) revealed a single peak corresponding to **Ce^IV^(Pc2)_2_** exhibiting the anticipated isotopic pattern and the absence of free ligands or triple-deckers. 

Following complexation, the carbazole protons are no longer equivalent due to restricted rotation along the C-N bond at room temperature. Consequently, the carbazole signals are split into two groups, as illustrated in Figure 7, with one part of the carbazole on the side of the Ce(IV) center while the second part is outside. It is known from the literature that the *in* signals are shifted downfield and the *out* signals are shifted upfield due to the strong ring current generated by the porphyrinoid ligands [35,36]. Consequently, the aromatic protons of the carbazole resonate can be divided into two groups, in and out, with signals for H_a_ at 7.12 ppm (in) and 5.23 ppm (out), for H_b_ at 8.05 ppm (in) and 5.97 ppm (out), and for H_c_ at 8.69 ppm (in) and 8.40 ppm (out). Additionally, the protons of the Pc ring resonate at 7.54 ppm (H_g_), 7.21 ppm (H_f_), and 6.62 ppm (H_e_). A similar effect is observed for the tert-butyl protons with two singlets at 1.86 and 1.02 ppm (Figure S7).

Increasing the temperature did not change the spectrum, indicating that the carbazole substituents cannot freely rotate, which is not surprising considering the X-ray structure. As this complex is prepared from two symmetrical **Pc2** ligands, we cannot be certain if there is a rotation of the Pc ring around its C_4_ symmetry axis, as the spectrum remains the same with or without rotation. To obtain such information, we investigated the rotation in a double-decker with comparable steric hindrance, prepared using two desymmetrized **Pc3** ligands. 

**Ce^IV^(Pc3)_2_** complexation was carried out by following the same strategy. The formation of the complex was confirmed by MALDI-TOF-MS with the expected molecular ion peaks and isotopic distribution. The ^1^H-NMR spectrum at room temperature was very complex due to the presence of many rotamers (Figure S12). Unfortunately, even at high temperature (140 °C), no changes have been observed (Figure S13). This indicates that the energy barrier is too large in this sterically overcrowded system, preventing any rotation around the pseudo-C_4_ symmetry axis.

#### 2.3.2. Heteroleptic Double-Decker **Ce^IV^(Pc)(Pc3)**

Since **Ce^IV^(Pc3)_2_** was unsuitable for studying rotational motions due to the tight engagement of the upper and lower decks, we prepared a heteroleptic Ce^IV^ double-decker complex with **Pc3** and the unfunctionalized **Pc**. Lowering the symmetry of the phthalocyanine ring is crucial for fine-tuning the physicochemical properties [30] but also to facilitate the tracking of rotation. There are only a few examples in the literature of heteroleptic double-decker complexes incorporating one desymmetrized **Pc** ring [7,37,38,39].

To prepare the heteroleptic double-decker complex, one equivalent of **H_2_Pc3** and one equivalent of **H_2_Pc** were reacted with Ce(acac)_3_·nH_2_O using microwave in o-DCB at 270 °C. This reaction led to a mixture of three compounds, with two homoleptic complexes, **Ce^IV^ (Pc3)_2_** and **Ce^IV^ (Pc)_2_**, and the desired complex, **Ce^IV^ (Pc)(Pc3)**, obtained with a yield of 37% after column chromatography. Their structures were confirmed by ^1^H-NMR and MS with the expected molecular ion peaks and isotopic distribution (Figure S18). 

### 2.4. 1H-NMR Studies of the Dynamic Internal Rotating Motions in ***Ce^IV^(Pc)(Pc3)***

The rotational flexibility of double-decker structures has been a subject of notable interest. Extensive studies employing variable-temperature ^1^H NMR have been conducted on lanthanoid and zirconium (IV) double-decker complexes [20,21]. In 2000, Aida and co-workers investigated this type of intramolecular rotation using chiral cerium(IV) and zirconium(IV) double-decker complexes of an ABAB porphyrin ring [40]. The ligand rotation in these double-decker complexes was largely influenced by the steric hindrance of the substituent and the central metal atom. To the best of our knowledge, the dynamic behavior of heteroleptic double-decker complexes comprising an A3B Pc ring with highly sterically hindered perpendicular substituents has not been reported.

The ^1^H-NMR spectrum of **Ce^IV^(Pc)(Pc3)** is very complex as revealed by the number of signals (Figure S15). As expected, the aromatic region of the spectrum contains a large number of well-resolved signals but also some overlapped and broad signals. Due to the low symmetry and the lack of rotation around the metal center, all the protons are inequivalent, even the four subunits of the unfunctionalized Pc. 

**Figure 8:**
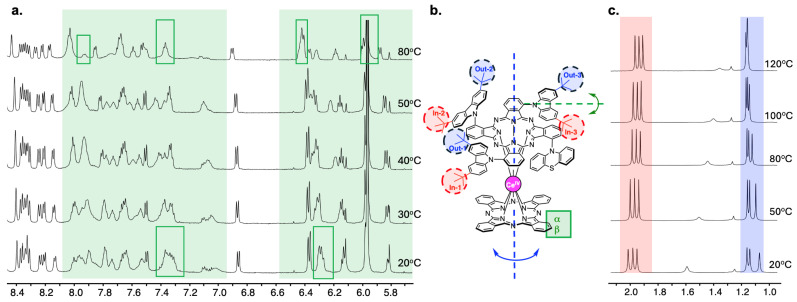
VT-^1^H-NMR spectra of **Ce^IV^(Pc)(Pc3)** in C_2_D_2_Cl_4_ (600 MHz): (**a**) In the aromatic region, the signals corresponding to the α and β protons (green region) are shifted and simplified at higher temperatures. (**b**) The molecular structure with the color code of the discussed protons; in red and blue are the *in* and *out tert*-butyl protons, and in green is the **Pc** protons. (**c**) In the aliphatic region, the signals corresponding to the *tert*-butyl protons are splitted in two groups. While the in signals are not changed, The blue and green arrows correspond to the rotation axis around the pseudo-C_4_ symmetry axis (blue-dotted axis) and the C-N bound axis between the phthalocyanine and carbazole (green-dotted axis).

The total integration of all aromatic signals fits well with the number of aromatic protons (54H) and the *tert*-Bu protons (also 54H). The *tert*-Bu signals are divided into two groups, with three singlets at around 1 ppm (*out*) and three singlets at around 2 ppm (*in*). Thus, variable-temperature NMR spectroscopy has been measured in the range of −20 to 120 °C (Figure S16) to see if the rotation processes around the axis shown in green and blue in Figure 8 can be investigated. Firstly, the *tert*-Bu signals are still divided into two groups (Figure 8c), even at high temperatures, which means the carbazole substituents cannot fully rotate around the C-N bond of the carbazole functionalization (green axis). They are just oscillating faster, inducing a shift for some singlets. A full and fast rotation would have given one set of three singlets without discrimination of the *in* and the *out tert*-butyl groups. 

The expected 18 signals for the carbazole sub-units and the 8 signals for the phenothiazine sub-unit were all identified (Figure S15). The remaining aromatic protons, therefore, arise from the α and β protons of the two Pc rings. Sixteen protons were sharp while twelve were shown to be broad, sometimes visible or overlapping with other signals. The signals correlating by group of three protons (from the ^1^H-^1^H COSY experiment) correspond to the functionalized Pc ring, and the signals correlating by group of four protons were assigned to the unfunctionalized Pc. The broad signals could be due to a slow exchange between different conformers due to the rotation of the ligands around the cerium ion [21]. A full and fast rotation would have given only one signal for each of the α and β protons. In our case, even at high temperature we can notice that the signals corresponding to the α and β protons are shifted and simplified (Figure 8a, green region) but remain as more than two signals, which means the unfunctionalized **Pc** ring is rotating faster at high temperature, but is still slower than the NMR timescale. 

## 3. Materials and Methods

### 3.1. General Informations

All reagents and solvents were purchased at the highest commercial quality available and used without further purification, unless otherwise stated. Anhydrous tetrahydrofuran, hydrochloric acid, and chloroform were purchased from FUJIFILM Wako Pure Chemical Corporation (Osaka, Japan). Lithium and Ce(acac)_3_·*n*H_2_O were purchased from Sigma Aldrich (St. Louis, MO, USA). Dichloromethane and phenothiazine were purchased from Nacalai tesque, Inc. (Kyoto, Japan). 3-fluorophthalonitrile and 3,6-di-*tert*- butyl-9H-carbazole were purchased from BLD Pharmatech Ltd. (Shangai, China). Aluminium(III) chloride was purchased from TCI (Tokyo, Japan). 

Silica gel column chromatography and thin-layer (TLC) chromatography were performed using Wakosil^®^ 60 and Merck silica gel 60 (F254) TLC plates, respectively. ^1^H and ^13^C NMR spectra were recorded on a JEOL JNM-ECA600 (600 MHz for ^1^H; 150 MHz for ^13^C) spectrometer, a JEOL JNM-ECZ500 (500 MHz for ^1^H; 125 MHz for ^13^C) spectrometer, or a JEOL JNM-ECX400P (400 MHz for ^1^H; 100 MHz for ^13^C) spectrometer at a constant temperature of 25 °C unless otherwise specified. Tetramethylsilane (TMS) was used as an internal reference for ^1^H and ^13^C-NMR measurements in CDCl_3_ and C_2_D_2_Cl_2_. A residual peak of a solvent was used as an internal reference for ^1^H-NMR measurements in CD_2_Cl_2_, o-DCB-d_4_, and DMSO-d_6_, and chemical shifts (*δ*) are reported in ppm. Coupling constants (*J*) are given in Hz and the following abbreviations are used to describe the signals: singlet (s); broad singlet (br. s); doublet (d); triplet (t); quadruplet (q); quintuplet (qt); and multiplet (m). Full assignments of ^1^H-NMR spectra were made with the assistance of COSY. The numbering system used for the assignment of signals is provided along with the corresponding spectra in the supporting information. The EI mass spectrometry was performed using JEOL AccuTOF JMS-T100LC. MALDI-TOF mass spectrometry was performed using a JEOL JMS-S3000 spectrometer. CEM Discover SP was used for reactions using a microwave irradiator. Single-crystal X-ray structure analysis was performed using Rigaku ValiMax RAPID (Rigaku, Tokyo, Japan).

### 3.2. Synthesis

#### 3.2.1. Homoleptic Cerium(IV) Double-Decker Complex **Ce^IV^(Pc2)_2_**

In a 10 mL microwave vial, **H_2_Pc2** (0.81 g, 1.6 eq., 500 µmol and Ce(acac)_3_·nH_2_O (0.17 g, 1 eq., 382 µmol) was mixed in 5 mL of o-DCB. N_2_ was purged into the mixture and the sample was irradiated with microwave at 270 °C for 3 cycles of 1 h. The reaction was monitored after each cycle by TLC in hexane/CH_2_Cl_2_ (7:3). After precipitation with MeOH and filtration, the collected solid was purified by column chromatography on silica eluted with CH_2_Cl_2_. The three stereoisomers give only one spot on TLC (Rf = 0.37 in hexane/CH_2_Cl_2_ 3:1). A green compound was further purified by recycling GPC (JAIGEL 2H-2.5H; eluent: CHCl_3_), followed by recrystallization with CHCl_3_ and MeOH to obtain **Ce^IV^(Pc2)_2_** in 70% yield (0.60 g). ^1^H-NMR (400 MHz, CDCl_3_): δ 8.69 (d, *J* = 1.2 Hz, 8H, c_in_), 8.40 (d, *J* = 2.0 Hz_,_ 8H, c_out_), 8.05 (dd, *J* = 1.6, 8.4 Hz, 8H, b_in_), 7.53 (d, *J* = 7.2 Hz, 8H, g), 7.19 (t, 8H, f), 7.12 (d, *J* = 8.4 Hz, 8H, a_in_), 6.61 (d, *J* = 7.6 Hz, 8H, e), 5.97 (dd, *J* = 1.6, 8.8 Hz, 8H, b_out_), 5.23 (d, *J* = 8.4 Hz, 8H, a_out_), 1.86 (s, 72H, d_in_), 1.02 (s, 72H, d_out_). ^13^C NMR (100MHz, CDCl_3_): 156.2, 154.2, 143.0, 142.1, 140.2, 139.6, 136.3, 132.6, 131.8, 129.9, 127.1, 124.1, 123.8, 122.3, 115.9, 115.1, 111.5, 111.2, 35.3, 34.4, 32.7, 31.8. UV-vis (CHCl_3_) λ_max_ (ε): 298 (159,090), 334 (110,460), 566 (23,360), 635 (sh, 29,440), 700 (113,230). HR-MS (MALDI-TOF-MS): m/z calculated for [Ce^IV^(Pc2)_2_]^+^ (C_224_H_216_N_24_Ce) 3381.6690; found 3381.6817. Elemental analysis: calcd. C_224_H_216_N_24_Ce (%): C(79.49), H(6.43), N(9.93); found (%): C(79.63), H(6.40), N(9.57).

#### 3.2.2. Homoleptic Cerium(IV) Double-Decker Complex **Ce^IV^(Pc3)_2_**

In a 10 mL microwave vial, **H_2_Pc3** (0.10 g, 1.6 eq., 65 mmol) and Ce(acac)_3_·nH_2_O (17 mg, 1 eq., 0.041 mmol) were mixed in 1 mL of o-DCB. After N_2_ was purged into the mixture, the solution was irradiated with microwave at 270 °C for 3 cycles of 1 h. The reaction was monitored after each cycle by TLC in hexane/CH_2_Cl_2_ (4:1). After precipitation with MeOH and filtration, the collected solid was purified by column chromatography on silica eluted with CH_2_Cl_2_. The first eluted green band was collected and was subjected to size-exclusion column chromatography (Biobeads SX-1, 4ø × 65 cm) with toluene. The dark blue compound in the first fraction corresponded to **Ce^IV^(Pc3)_2_** in 39% yield (51.5 mg) along with 28% (18 mg) of **H_2_Pc3** in a second fraction (dark green). ^1^H-NMR: δ 8.89–5.27 (m), 4.68–4.32 (m), 2.11–1.74 (m, d_in_), 1.28–0.95 (m, d_out_). A complicated ^1^H-NMR spectrum was obtained due to many conformers. UV-vis (CHCl_3_) λ_max_ (ε): 299 (91,380), 335 (76,310), 682 (65,190). HR-MS (MALDI-TOF-MS): m/z calculated for [Ce^IV^(Pc3)_2_]^+^ 3221.3627; found 3221.3639.

#### 3.2.3. Heteroleptic Cerium(IV) Double-Decker Complex **Ce^IV^(Pc)(Pc3)**

In a 10 mL microwave vial, **H_2_Pc3** (150 mg, 0.8 eq., 972 µmol) and Ce(acac)_3_·nH_2_O (53 mg, 1.0 eq., 121 µmol) and Ce(acac)_3_·nH_2_O (53 mg, 1.0 eq., 121 µmol) were mixed in 2 mL of o-DCB. After N_2_ was purged into the mixture, the solution was irradiated with microwave at 270 °C for 3 cycles of 1 h. The reaction was monitored after each cycle by TLC in hexane/CH_2_Cl_2_ (1:1) and MALDI-TOF-MS. After precipitation with MeOH and filtration, the collected solid was purified by column chromatography on silica eluted with CH_2_Cl_2_. The first eluted green band was collected and concentrated under reduced pressure. The compound with an Rf value of 0.26 was found to be the desired compound but a second column chromatography was necessary to purify it (SiO_2_, hexane/CH_2_Cl_2_ 1:1). Pure **Ce^IV^(Pc)(Pc3)** was obtained with a yield of 37% (47.1 mg). ^1^H-NMR (600 MHz, CDCl_3_): δ 8.82 (d, *J* = 1.2 Hz, 1H, c_in_), 8.79 (d, *J* = 1.2 Hz, 1H, c_in_), 8.66 (d, *J* = 8.4 Hz, 1H, a_in_), 8.61(d, *J* = 8.4 Hz, 1H, a_in_), 8.63 (s, 1H, c_in_), 8.48 (s, 1H, c_out_) 8.44 (d, *J* = 1.2 Hz, 1H, c_out_), 8.49 (s, 1H, αPc3), 8.35 (d, *J* = 1.2 Hz, 1H, c_out_), 8.34 (d, *J* = 1.8 Hz, 1H, b_in_), 8.32 (s, 1H, a_in_), 8.30 (dd, *J* = 9.0Hz, 1.8 Hz, 1H, b_in_), 8.23 (d, *J* = 7.2 Hz, 1H, βPc3), 8.15–8.11 (m, 2H, b_in_, αPc), 7.98 (d, *J* = 7.2 Hz, 1H, βPc3), 7.89 (t, *J* = 6.6 Hz, 1H, βPc3), 7.62–7.58 (m, 2H, βPc), 7.51 (t, *J* = 7.2 Hz, 1H, βPc3), 7.43–7.33 (m, 2H, a_out_), 7.36 (b, 1H, αPc), 7.28–7.22 (m, 1H, βPc), 7.24–7.28 (m, 1H, b_out_), 6.82 (d, *J* = 8.4 Hz, 1H, k_out_), 6.39 (d, *J* = 9.0 Hz, 1H, h_in_), 6.35 (d, *J* = 8.4 Hz, 1H, k_in_), 6.28 (d, *J* = 8.4 Hz, 1H, b_out_), 6.24 (d, *J* = 7.2 Hz, 1H, αPc), 6.19 (d, *J* = 6.6 Hz, 1H, αPc3), 6.10 (t, *J* = 7.8 Hz, 1H, j_out_), 5.94 (d, *J* = 7.2 Hz, 1H, a_out_), 5.94–5.88 (m, 2H, i,j_out_), 5,44 (t, *J* = 7.8 Hz, 1H, i_in_), 5.71 (d, *J* = 8.4 Hz, 1H, b_in_), 4.63 (d, *J* = 9.0 Hz, 1H, h_out_), 2.03 (s, 9H, ^t^Bu_in_), 2.00 (s, 9H, ^t^Bu_in_), 1.98 (s, 9H, ^t^Bu_in_), 1.21 (s, 9H, ^t^Bu_out_), 1.17 (s, 9H, ^t^Bu_out_), 1.15 (s, 9H, ^t^Bu_out_). ^13^C NMR (150 MHz, CD_2_Cl_2_): 154.2, 154.1, 153.9, 153.6, 151.4, 151.2, 151.0, 150.7, 144.3, 143.4, 143.3, 143.1, 142.9, 142.1, 141.9, 141.1, 141.0, 141.0, 140.8, 138.8, 138.3, 138.0, 134.5, 133.1, 132.8, 132.5, 131.2, 131.1, 131.0, 130.9, 130.7, 130.5, 130.2, 127.4, 126.7, 126.0, 125.7, 124.7, 124.6, 124.3, 124.2, 124.1, 124.0, 123.7, 123.3, 121.8, 121.3, 120.8, 120.2, 119.7, 119.2, 118.7, 118.1, 117.6, 117.1, 116.6, 116.1, 115.5, 115.0, 114.5, 114.0, 113.4, 112.9, 112.4, 111.9, 111.3. UV-vis (CHCl_3_) λ_max_ (ε): 298 (63,780), 571 (26,850), 668 (27,330), 740 (13,350). HR-MS (MALDI-TOF-MS): m/z calculated for [**Ce^IV^(Pc)(Pc3)**]^+^ 2192.7836; found 2192.7835.

## 4. Conclusions

In summary, three highly sterically hindered double-decker complexes have been prepared with phthalocyanines functionalized at the α-position despite their high steric hindrance. **Ce^IV^(Pc2)_2_** is a homoleptic complex of a tetrasubstituted Pc with carbazole and **Ce^IV^(Pc3)_2_** is a homoleptic complex of a desymmetrized Pc with three carbazoles and one phenothiazine. Since the rotative motions were blocked even at high temperatures in these homoleptic complexes, a heteroleptic **Ce^IV^(Pc)(Pc3)** complex was also prepared with one unfunctionalized ligand.

The dynamic behaviors of the **Ce^IV^(Pc3)_2_** and **Ce^IV^(Pc)(Pc3)** complexes were analyzed by VT-NMR from −20 °C to 140 °C. In the case of **Ce^IV^(Pc3)_2_**, no change in the spectrum was observed, illustrating the too-high steric hindrance preventing any rotation from occurring. In the case of **Ce^IV^(Pc)(Pc3)**, we demonstrated that rotation along the pseudo-C_4_ symmetry axis can be switched on by heating the solution. The observed rotation is slow at room temperature, but as the temperature increases, some signals corresponding to the α- and β- protons of the **Pc** ligands appear as well-resolved signals. Additionally, the carbazole substituents do not fully rotate even at higher temperatures, but their faster oscillation allows the unfunctionalized **Pc** fragment to rotate, which was not possible in the case of the homoleptic complexes. We are now working on modifying such complexes to deposit them on a metallic surface to build a train of gears and observe the intermolecular transfer of rotating motions. 

## Data Availability

The data presented in this study are available on request from the corresponding author.

## Supplementary Materials

The following supporting information can be downloaded at https://www.mdpi.com/article/10.3390/molecules29040888/s1: Figure S1: ^1^H-NMR spectrum of **H_2_Pc1**; Figure S2: ^13^C NMR spectrum of **H_2_Pc1**; Figure S3: HR-MALDI-TOF MS for **H_2_Pc1**; Figure S4: ^1^H-NMR of **H_2_Pc3**; Figure S5: ^13^C-NMR spectrum of **H_2_Pc3**; Figure S6: HR-MALDI-TOF MS for **H_2_Pc3**; Figure S7. ^1^H-NMR spectrum of **Ce^IV^(Pc2)_2_**; Figure S8: ^1^H-NMR spectra of **Ce^IV^(Pc2)_2_** in C_2_D_2_Cl_4_ at 20 °C and 100 °C; Figure S9: COSY spectrum of **Ce^IV^(Pc2)_2_**; Figure S10: ^13^C-NMR spectrum of **Ce^IV^(Pc2)_2_** (CDCl_3_, 100MHz); Figure S11: HR-MALDI-TOF MS for **Ce^IV^(Pc2)_2_**; Figure S12: ^1^H-NMR spectrum of **Ce^IV^(Pc3)_2_**; Figure S13: ^1^H-NMR spectra of **Ce^IV^(Pc3)_2_** at 25 °C and 140 °C; Figure S14: HR-MALDI-TOF MS for **Ce^IV^(Pc3)_2_**; Figure S15: ^1^H-NMR spectrum in C_2_D_2_Cl_4_ (600 MHz) of **Ce^IV^(Pc)(Pc3)**; Figure S16: Full VT-^1^H-NMR spectra of **Ce^IV^(Pc)(Pc3)** from -20 to 120 °C; Figure S17: ^13^C-NMR spectrum of **Ce^IV^(Pc)(Pc3**); Figure S18: HR-MALDI-TOF MS for **Ce^IV^(Pc)(Pc3)**; Figure S19: Picture of the crystal of **Ce^IV^(Pc2)_2_**; Figure S20: ORTEP representation, table of crystal data and structure refinement for complex of **Ce^IV^(Pc2)_2_**. Additional experimental section with the synthesis of **H_2_Pc1**, **H_2_Pc2**, and **H_2_Pc3**; crystallographic data can be obtained free of charge from the Cambridge Crystallographic Data Centre via https://www.ccdc.cam.ac.uk/structures/ (accessed on 14 February 2024). CCDC deposit numbers for double-decker complex **Ce^IV^(Pc2)_2_**: 2324826.
