# Supplementary material for: Synthesis and Dynamic Behavior of Ce(IV) Double-Decker Complexes of Sterically Hindered Phthalocyanines
Source: Molecules. 2024 Feb 17;29(4):888. doi: 10.3390/molecules29040888 (PMC10891708; doi:10.3390/molecules29040888)
Supplement: Supplementary file 1 [file molecules-29-00888-s001.zip › molecules-2860874-supplementary.pdf]

Supplementary Information

Synthesis and Dynamic behavior of Ce(IV) Double-Decker Complexes of Sterically-hindered Phthalocyanines

Jeevithra Dewi Subramaniam [1‡], Toshio Nishino [1‡], Kazuma Yasuhara [1,2] and Gwénaël Rapenne[1,3,*]

[1] Division of Materials Science, Nara Institute of Science and Technology, 8916-5 Takayama, Ikoma, 630-0192, Japan
[2] Center for Digital Green-innovation, Nara Institute of Science and Technology, 8916-5 Takayama-cho, Ikoma, 630-0192, Japan
[3] CEMES, Université de Toulouse, CNRS, 29, rue Marvig, 31055 Toulouse, France
‡ Equal contributors

Table of content

I. Additional experimental section

1. Phthalocyanine $\mathbf{H_2Pc1}$ page S2
2. Phthalocyanine $\mathbf{H_2Pc2}$ page S3
3. Phthalocyanine $\mathbf{H_2Pc3}$ page S4

II. ^{1}H-NMR, ^{13}C-NMR and HR Mass spectra

1. Phthalocyanine $\mathbf{H_2Pc1}$ page S5
2. Phthalocyanine $\mathbf{H_2Pc3}$ page S6
3. Homoleptic double decker complex $\mathbf{Ce^{IV}(Pc2)_2}$ page S8
4. Homoleptic double decker complex $\mathbf{Ce^{IV}(Pc3)_2}$ page S11
5. Heteroleptic double decker complex $\mathbf{Ce^{IV}(Pc)(Pc3)}$ page S12

III. Crystallographic data

1. Experimental details of single crystal X-ray analysis of complex $\mathbf{Ce^{IV}(Pc2)_2}$ page S15
2. Molecular structure of complex $\mathbf{Ce^{IV}(Pc2)_2}$ page S15
3. Crystal data and structure refinement for complex $\mathbf{Ce^{IV}(Pc2)_2}$ page S16

I. Additional experimental section

I.1. Synthetic route to H_2Pc1 :

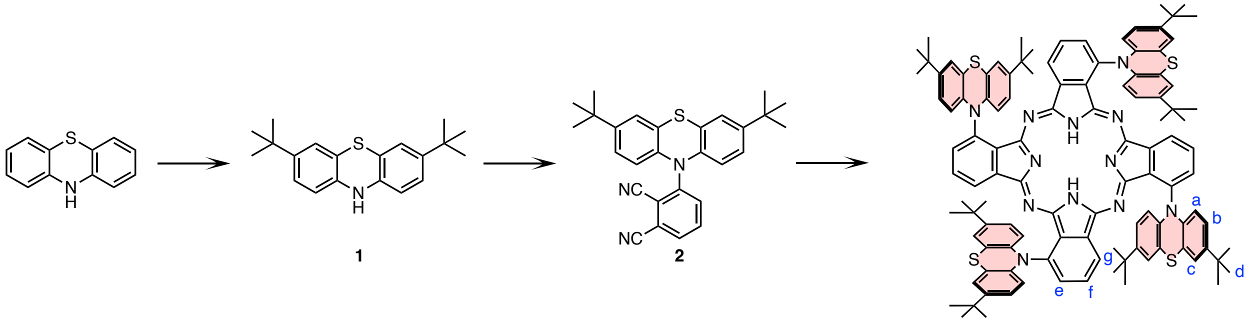

H_2Pc1

3,6-di-tert-butyl phenothiazine (1)

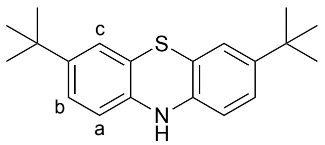

Compound **1** was synthesized by following a modified reported procedure [G. Sych, R. Pashazadeh, Y. Danyliv, O. Bezvikonnyi, D. Volyniuk, A. Lazauskas, J. V. Grazulevicius, *Chem. – Eur. J.* **2021**, *27*, 2826–2836]. In a 2-necked round bottom flask, tert-butyl chloride (21.29 g, 5 eq., 230 mmol) was added dropwise to a vigorously stirred suspension of phenothiazine (9.17 g, 1 eq., 46 mmol) and anhydrous aluminium chloride (12.88g, 2.1 eq., 97 mmol) in 100mL of CH_2Cl_2 under N_2 at 0°C. After the addition, the reaction mixture was stirred at 0 °C for 1 h. Then, the red mixture was quenched with water and extracted with CH_2Cl_2 three times. The organic layer washed with saturated sodium bicarbonate, saturated sodium dithionite solution, and dried with sodium sulfate. The solvent was then evaporated, and the crude was purified by column chromatography (CH_2Cl_2). **1** was obtained with a 79% yield (11.25 g) as a colorless solid. ^{1}H-NMR of compound **1** was in agreement with literature.

^{1}H-NMR (400 MHz, DMSO-d_6): δ 8.39 (s, NH, 1H), 6.97 (dd, J = 2.4, 8.8 Hz, H_b, 2H), 6.87 (d, J = 2.0 Hz, H_c, 2H), 6.60 (d, J = 8.4 Hz, H_a, 2H), 1.18 (s, tert-Bu, 18H).

3-(3,6-di-tert-butyl-10H-phenothiazinyl)phthalonitrile (2)

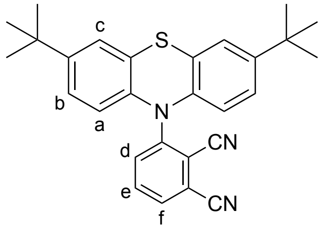

In a two-necked round bottom flask, 30mL of anhydrous DMF was added to NaH (50% in oil, 0.80 g, 1.5 eq., 17 mmol) and **1** (3.46 g, 1.0 eq., 11 mmol) under N_2. The suspension was stirred at 0°C using an ice bath for 3 h. 3-fluorophthalonitrile (1.61 g, 1 eq., 11 mmol) in 170 mL of anhydrous DMF was added dropwise to the reaction mixture and stirred overnight at room temperature. Ice cold water was added to the reaction mixture to precipitate a greenish oily solid. The crude was extracted with ethyl acetate and then washed with water and saturated aqueous sodium chloride. The organic phase was then evaporated and purified by column chromatography with hexane:ethyl acetate (7:3). Compound **2** was obtained as a yellow solid with a yield of 79% **(**3.81 g).

^{1}H-NMR (400 MHz, DMSO-d_6): δ 8.35 (dd, J = 2.4, 6.8 Hz, H_d, 1H), 8.19 (m, H_{e-f}, 2H), 7.12 (d, J = 2.4 Hz, H_c, 2H), 6.96 (dd, J = 2.0, 8.8 Hz, H_b, 2H), 6.05 (d, J = 8.8 Hz, H_a, 2H), 1.21 (s, tert-Bu,18H).

1,8,15,22-tetrakis[N-(3,6-di-tert-butylphenothiazine)]phthalocyanine (H_2Pc1)

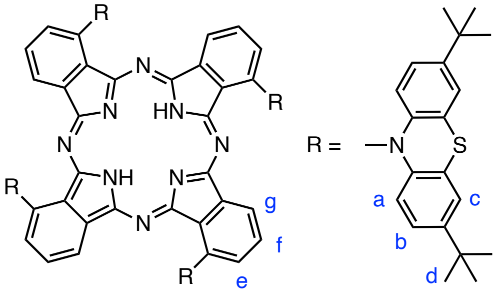

In a two-necked round bottom flask, Li (0.096 g, 2 eq., 13.71 mmol) was added to n-pentanol (55 mL) under inert atmosphere and the solution was heated at 130°C until total dissolution of Li. The reaction mixture was then cooled to room temperature and **2** (3.00 g, 1 eq., 6.86 mmol) was added into the reaction mixture. The solution was refluxed for 6 h to give a dark green solution which was neutralized with concentrated HCl before to be diluted with MeOH. After centrifugation of the resultant mixture, a fine green solid was collected. Purification of the crude by reprecipitation from $CHCl_3$:MeOH (1:2) yielded 45% (1.54g) of the metal-free phthalocyanine **H_2Pc1**.

^{1}H-NMR (400MHz, $CDCl_3$): δ 9.12 (d, J = 8.0 Hz, H_f, 4H), 8.25 (t, J = 8.0 Hz, H_e,4H), 8.11 (d, J = 7.6 Hz, H_d 4H), 7.28 (d, J = X.X Hz, 8H), 6.45 (dd, J = 2.4, 9.2 Hz, H_b, 8H), 6.09 (d, J = 8.8 Hz, H_c, 8H), 1.16 (s, ^{t}Bu, 72H), -0.61 (s, 2H, NH).

^{13}C-NMR (100 MHz, $CDCl_3$): 144.9, 142.0, 136.6, 134.4, 132.6, 124.9, 123.8, 123.5, 118.3, 115.2, 33.9, 31.2.

UV-vis ($CHCl_3$): λ_{max} (ε) : 336 (54660),609 (sh, 16770), 667 (52470), 704 (60960).

HR-MS (MALDI-TOF-MS): m/z calculated for [**H_2Pc1**]$^+$ 1750.7854; found 1750.7859

I.2. 1,8,15,22-tetrakis[N-(3,6-di-tert-butycalbazole)amino]phthalocyanine (H_2Pc2)

H_2Pc2 as been synthesized following a described procedure [S. Yamamoto, K. Kuribayashi, T. N. Murakami, E. Kwon, M. J. Stillman, N. Kobayashi, H. Segawa, M. Kimura, *Chem. Eur. J.* **2017**, *23*, 15446–15454.].

I.3. 1,8,15-tri[N-(3,6-di-tert-butyl carbazolyl)-22-phenothiazinyl]phthalocyanine (H_2Pc3)

3-(10H-Phenothiazin-10-yl)phthalonitrile

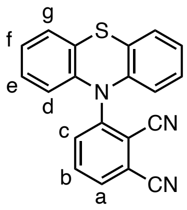

NaH in oil (60%, 2.47 g, 62 mmol) was added to the solution of phenothiazine (10.02 g, 50 mmol) in anhydrous DMF (100 mL) at 0 °C. The resulting mixture was stirred for 20 min, and then 3-fluorophthalonitrile (7.41 g, 51 mmol) was added. The reaction mixture was stirred for 1 hr at 0°C. H_2O (150 mL) was added to quench the reaction. The generated precipitate was filtrated and washed with H_2O and hexane to obtain the title compound as a yellow solid with a yield of 95% (15.49 g).

^{1}H-NMR (400 MHz, $CDCl_3$): δ 8.00–7.94 (m, 3H, $H_{a, b, c}$), 7.13–7.11 (m, 2H, H_g), 6.95–6.91 (m, 4H, $H_{e, f}$), 6.01–6.08 (m, 2H, H_d).

^{13}C NMR (150MHz, $CDCl_3$): δ 145.7, 142.1, 138.0, 135.0, 133.1, 127.5, 127.1, 124.0, 122.1, 119.5, 119.2, 115.8, 114.7, 112.7.

1,8,15-tri[N-(3,6-di-tert-butyl carbazolyl)-22-phenothiazinyl]phthalocyanine (H_2Pc3)

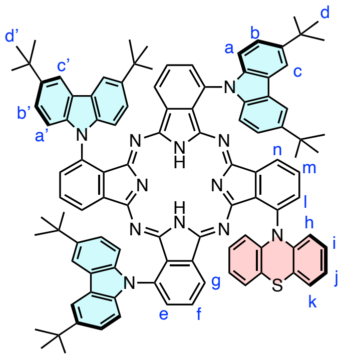

In a two-necked round bottom flask, Li (0.061 g, 8 eq., 8.8 mmol) was added to n-pentanol (20 mL) under inert atmosphere and the solution was refluxed until dissolution of Li. After cooling the reaction mixture to room temperature, 3-(3,6-di-tert-butyl-9H-carbazol-9-yl)phthalonitrile (1.23 g, 3 eq., 3.0 mmol) and 3-(10H-phenothiazin-10-yl)phthalonitrile (0.33 g, 1 eq., 1.0 mmol) were added into the reaction mixture. The solution was then refluxed 7 h and cooled to room temperature. The dark green solution was neutralized with concentrated HCl and diluted with MeOH. The resultant precipitate was collected by filtration and washed with MeOH. The crude compound was purified by column chromatography on silica (hexane/CH_2Cl_2 8:2) twice to obtain **H_2Pc3** with a yield of 22% (0.35 g).

^{1}H-NMR (400 MHz, $CDCl_3$): δ 9.24 (d, J = 6.6 Hz, 1H, H_n), 8.56 (d, J = 1.2 Hz, 2H, H_c), 8.55 (d, J = 1.8 Hz, 2H, H_c), 8.54 (d, J = 1.8 Hz, 2H, H_c), 8.27-8.23 (m, 2H, $H_{l,m}$), 8.17-8.13 (m, 2H, H_g), 8.00-7.97 (m, 4H, $H_{g,f}$), 7.53 – 7.50 (m, 3H, H_e), 7.37-7.21 (m, H_a, H_b, H_k), 6.70 (, J = 7.2 Hz, 2H, H_j), 6.42 (t, J = 8.4 Hz, 2H, H_i), 6.13 (d, J = 8.4 Hz, 2H, H_h), 1.54 (s, 18H, H_d), 1.53 (s, 18H, H_d), 1.51 (s, 18H, H_d).

^{13}C NMR (150MHz, $CDCl_3$): 144.2, 142.81, 140.7, 140.6, 140.5, 135.9, 133.7, 133.4, 133.3, 133.2, 132.3, 131.3, 130.9, 130.8, 130.7, 130.6, 126.8, 126.4, 124.5, 123.9, 123.8, 123.8, 123.6, 123.5, 123.4, 122.1, 118.6, 116.2, 116.2, 115.6, 110.9, 110.8, 110.7, 34.9, 34.8, 32.2, 32.1, 31.6.

UV-vis ($CHCl_3$): λ_{max} (ε) : 298 (54850) , 344 (55860) ,621 (sh, 18190) ,650 (sh, 25640), 718 (87100).

HR-MS (MALDI-TOF-MS): m/z calculated for [**H_2Pc3**]$^+$ 1542.7440; found 1542.7445.

II. ^{1}H-NMR, ^{13}C-NMR and Mass spectra

II.1. Phthalocyanine H_2Pc1

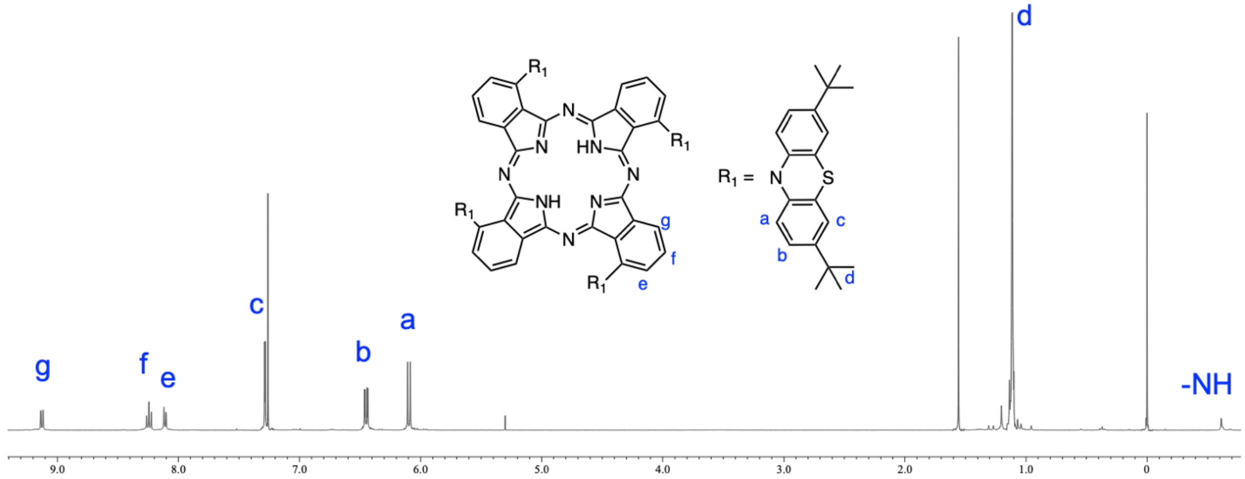

Figure S1. ^{1}H-NMR spectrum of $\mathbf{H_2Pc1}$ ($CDCl_3$, 400MHz)

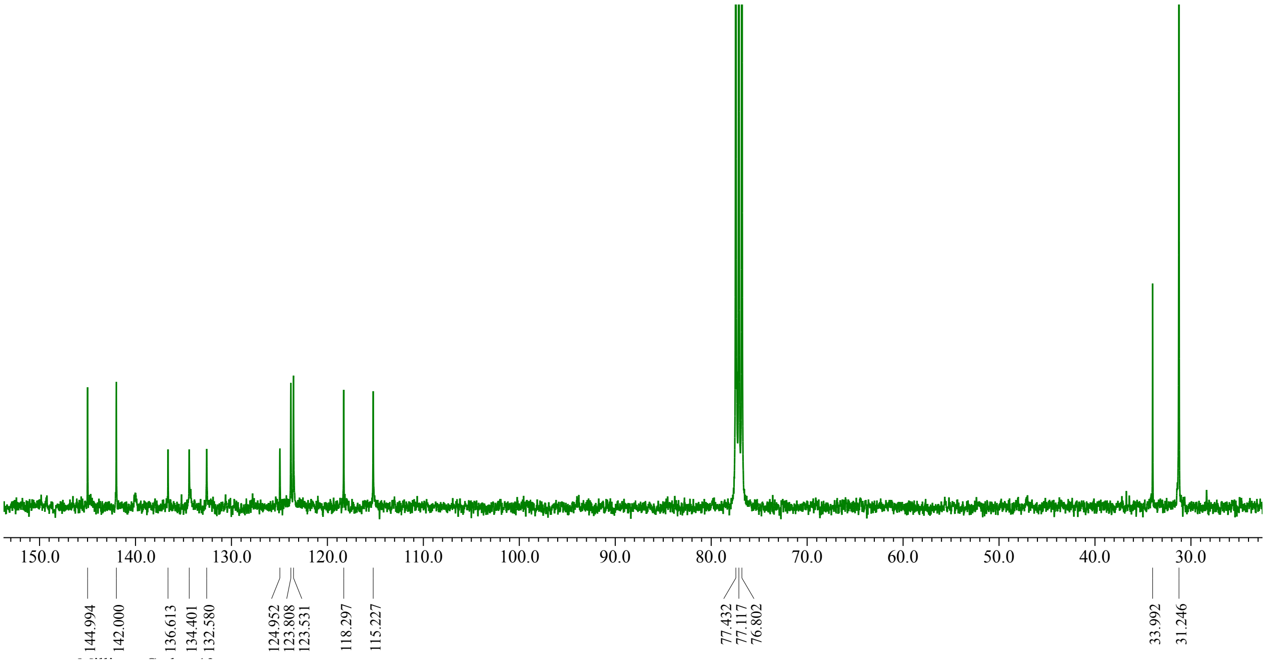

Figure S2. ^{13}C NMR spectrum of $\mathbf{H_2Pc1}$ ($CDCl_3$,100MHz)

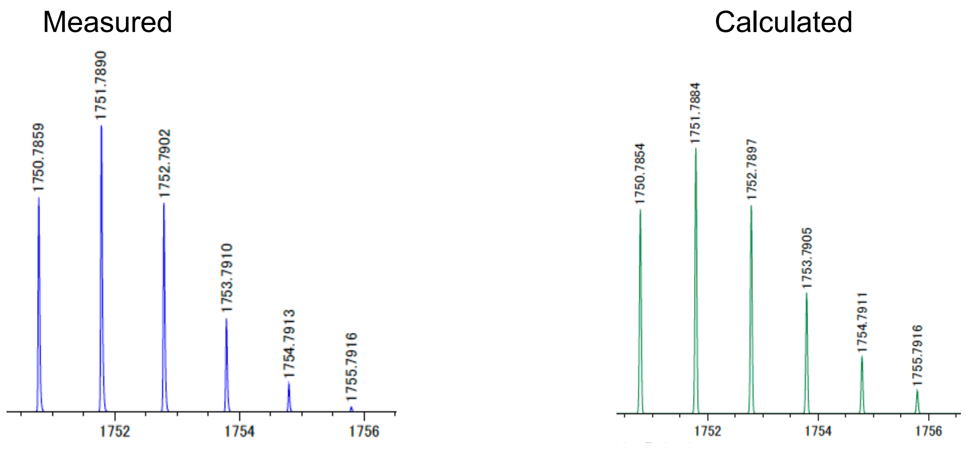

Figure S3. HR-MALDI-TOF MS for **H_2Pc1.**

II.2. Phthalocyanine H_2Pc3

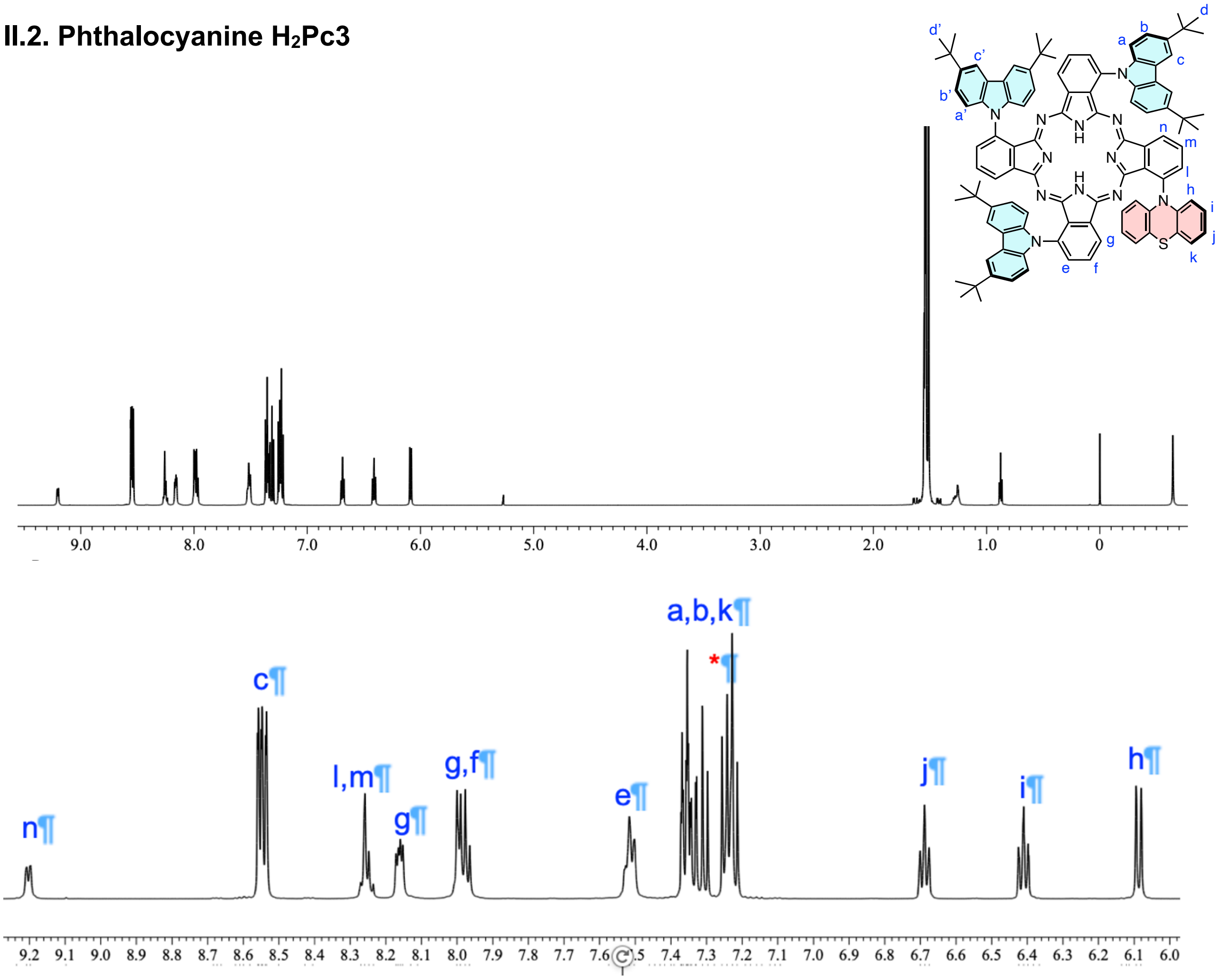

Figure S4. ^{1}H-NMR of **H_2Pc3** ($CDCl_3$, 600MHz).

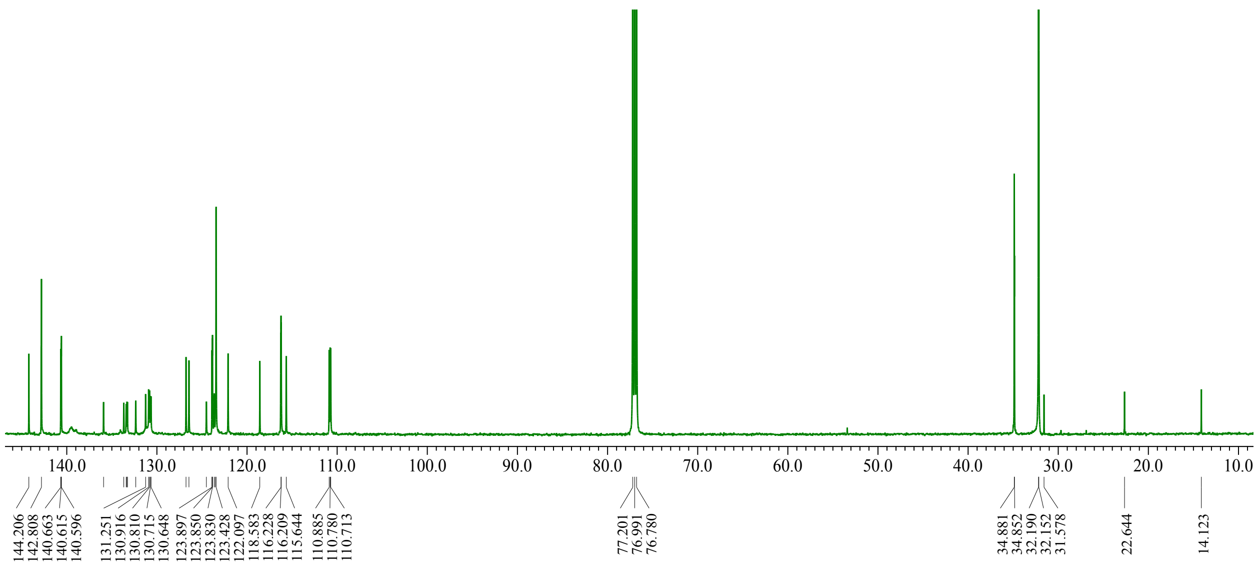

Figure S5. ^{13}C-NMR spectrum of **H_2Pc3** ($CDCl_3$, 150MHz)

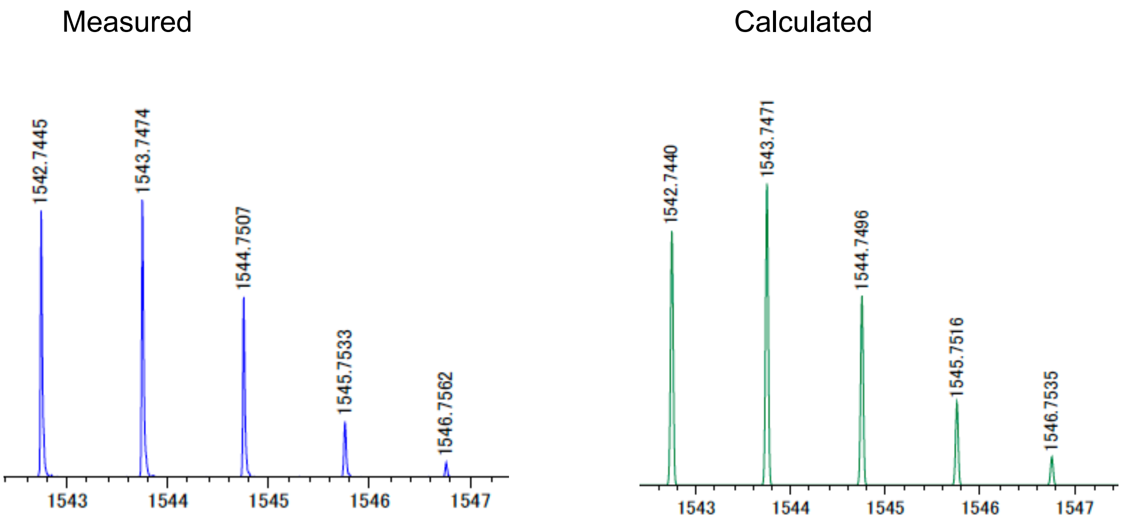

Figure S6. HR-MALDI-TOF MS for **H_2Pc3.**

II.3. Homoleptic double decker complex $Ce^{IV}(Pc2)_2$

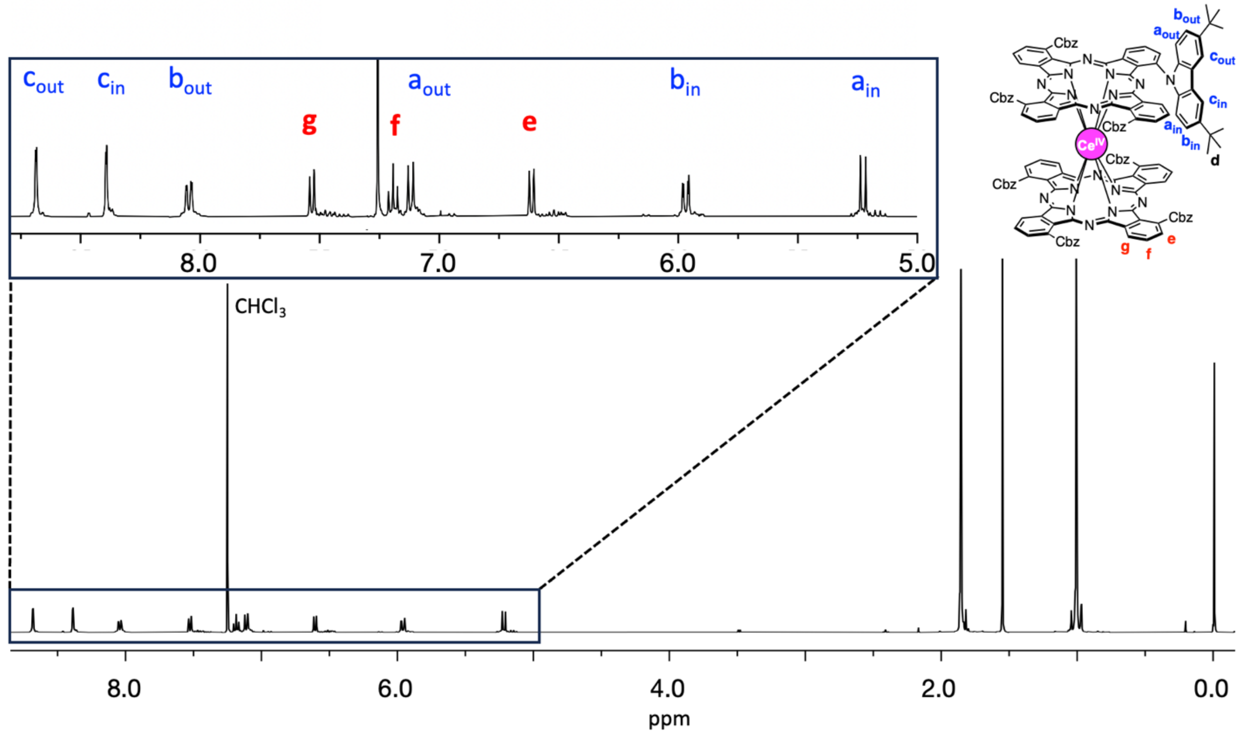

Figure S7. ^{1}H-NMR spectrum of **$Ce^{IV}(Pc2)_2$** ($CDCl_3$, 400MHz).

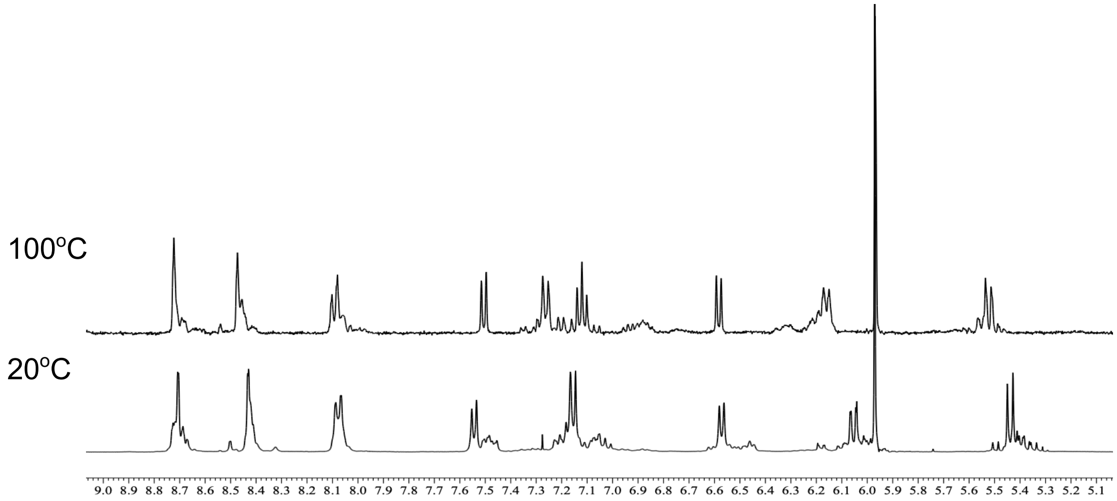

Figure S8. ^{1}H-NMR spectra of **$Ce^{IV}(Pc2)_2$** in $C_2D_2Cl_4$ at 20°C (bottom) and 100°C (top).

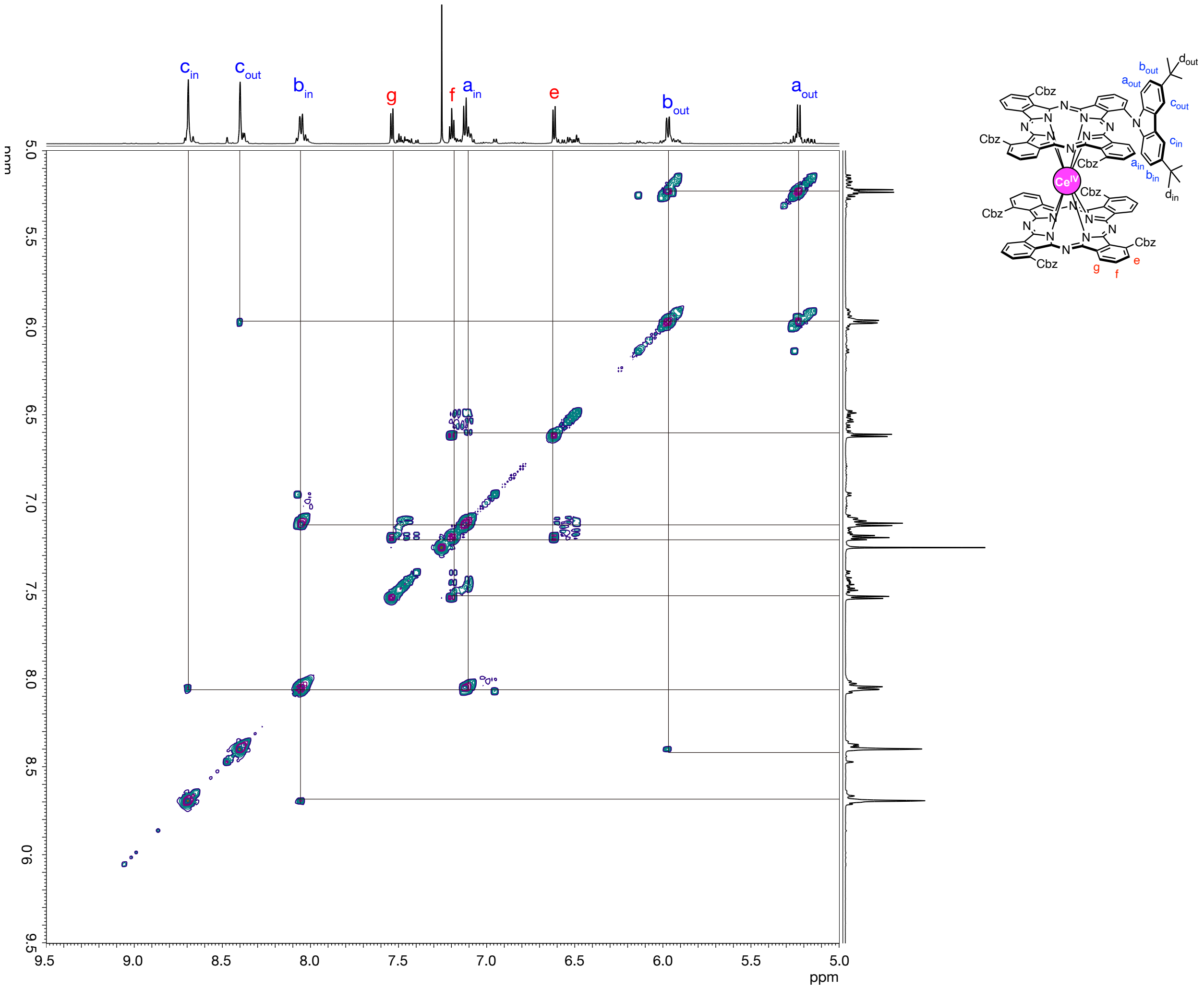

Figure S9. COSY spectrum of **Ce^{IV}(Pc2)$_2$** in $CDCl_3$.

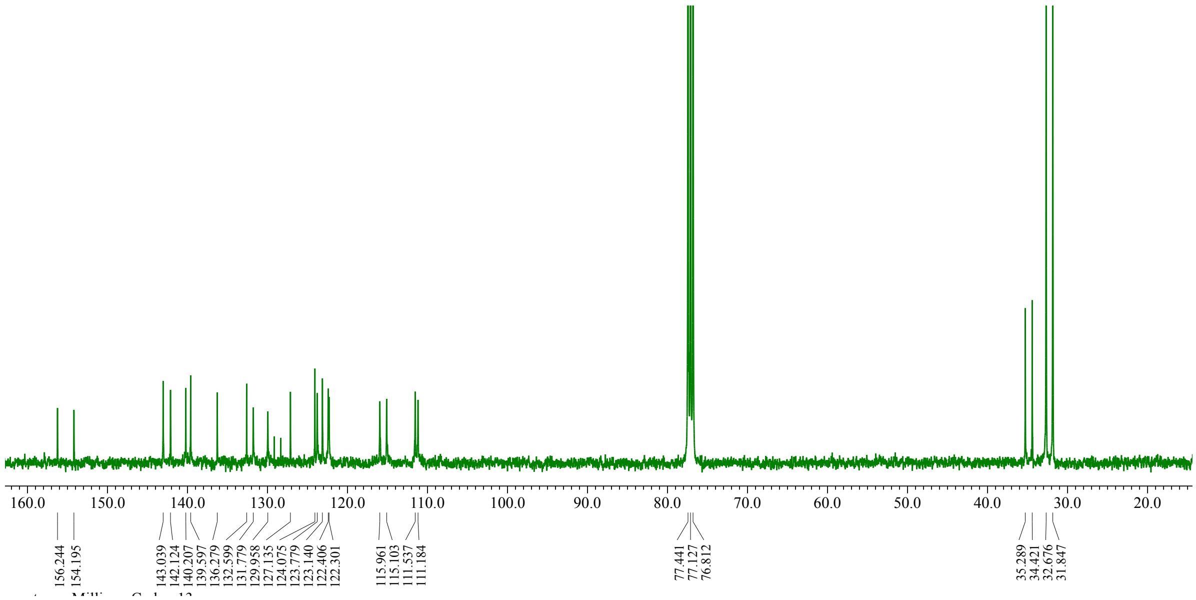

Figure S10. ^{13}C-NMR spectrum of **Ce^{IV}(Pc2)$_2$** ($CDCl_3$, 100MHz)

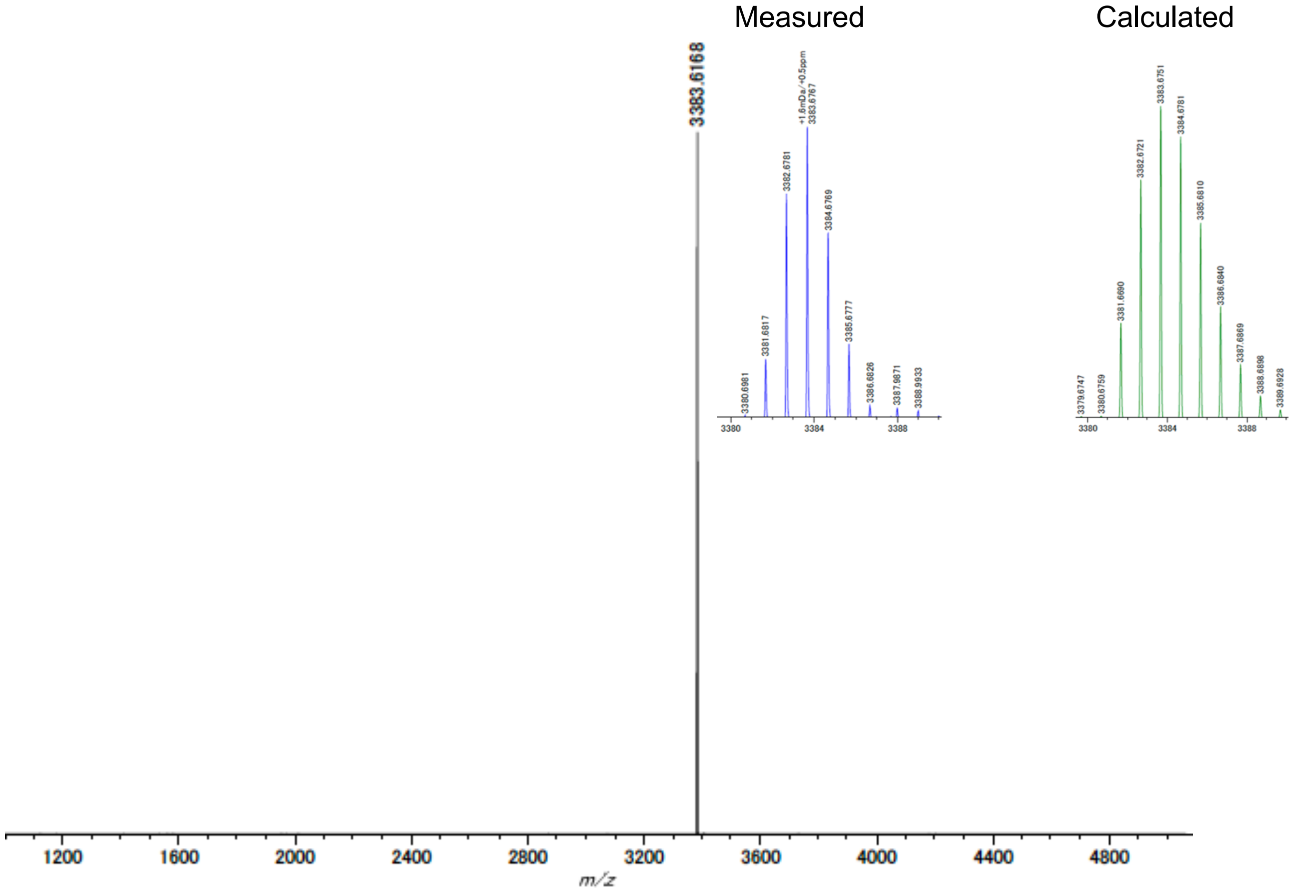

Figure S11. HR-MALDI-TOF MS for **Ce^{IV}(Pc2)$_2$**.

II.4. Homoleptic double decker complex $Ce^{IV}(Pc3)_2$

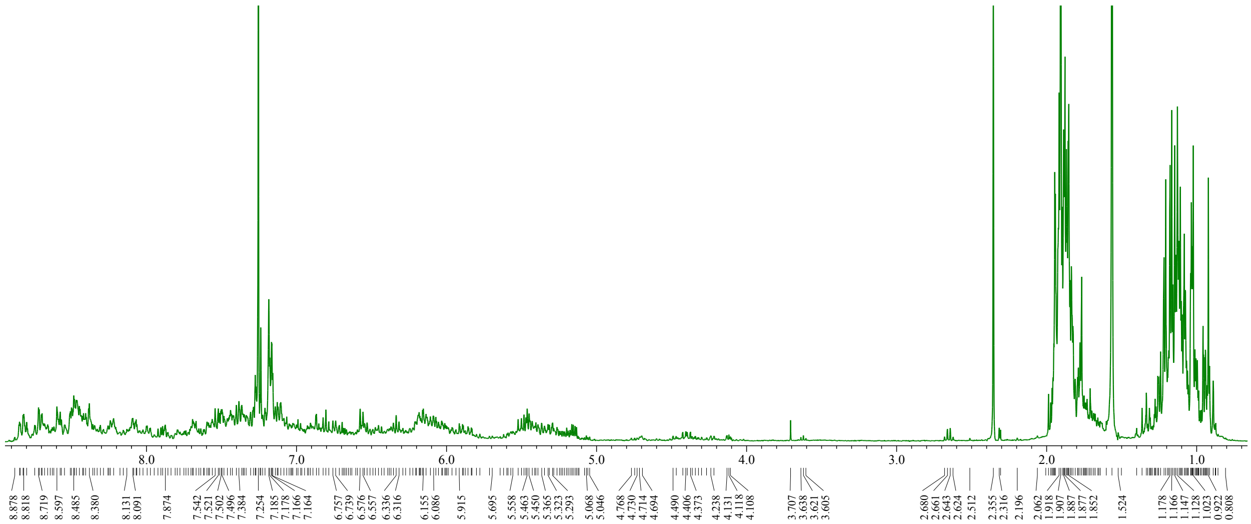

Figure S12. ^{1}H-NMR spectrum of **$Ce^{IV}(Pc3)_2$** ($CDCl_3$, 400MHz).

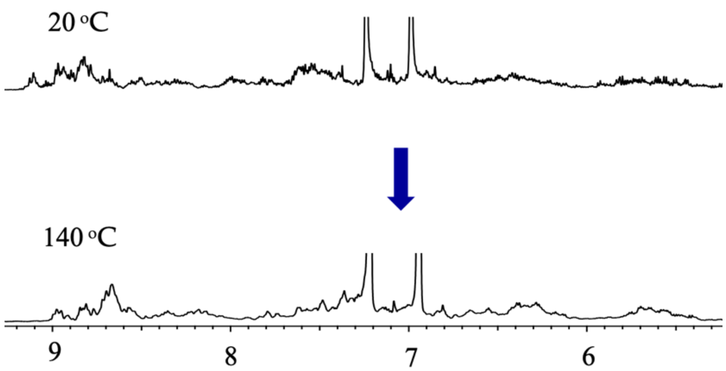

Figure S13. ^{1}H-NMR spectra of **$Ce^{IV}(Pc3)_2$** in *o*-DCB-*d4* at 25°C and 140°C.

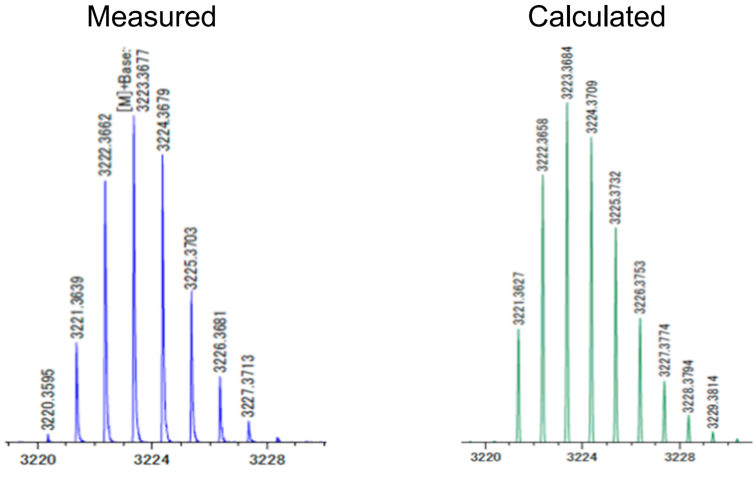

Figure S14. HR-MALDI-TOF MS for **$Ce^{IV}(Pc3)_2$.**

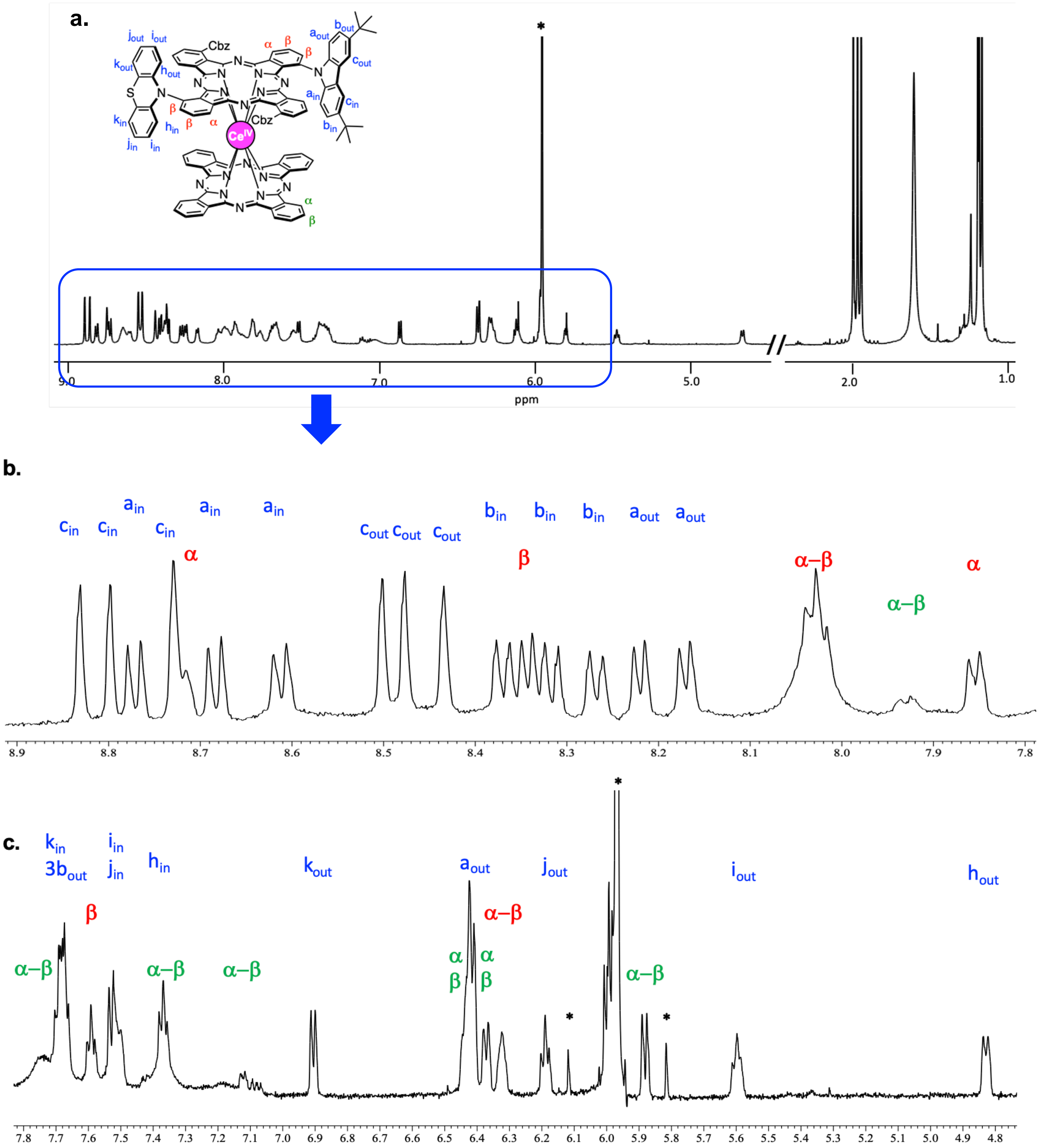

Figure S15. ^{1}H-NMR spectrum in $C_2D_2Cl_4$ (600 MHz) of **Ce^{IV}(Pc)(Pc3)**. (a) Full spectrum and molecular structure with labelling of protons. (b) Spectrum from 7.8 to 8.9 ppm (c) Spectrum from 4.8 to 7.8 ppm. Peak with stars (*) at 6 ppm and around coresponds to the residual C_2HDCl_4 and its ^{13}C satelites.

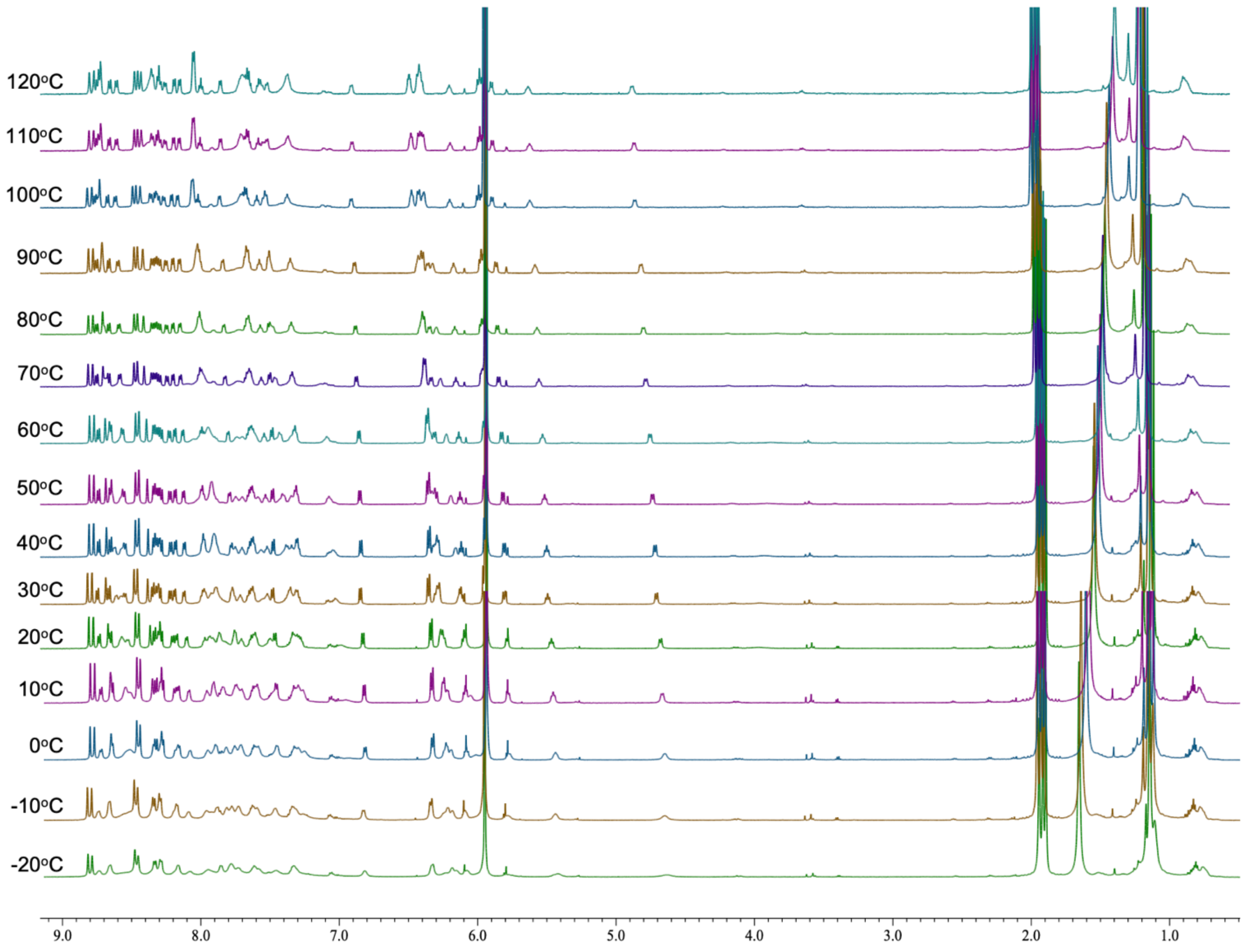

Figure S16. Full VT-^{1}H-NMR spectra of **Ce^{IV}(Pc)(Pc3)** in $C_2D_2Cl_4$ (600MHz) from -20 to 120°C. Peak at 6 ppm coresponds to the residual C_2HDCl_4.

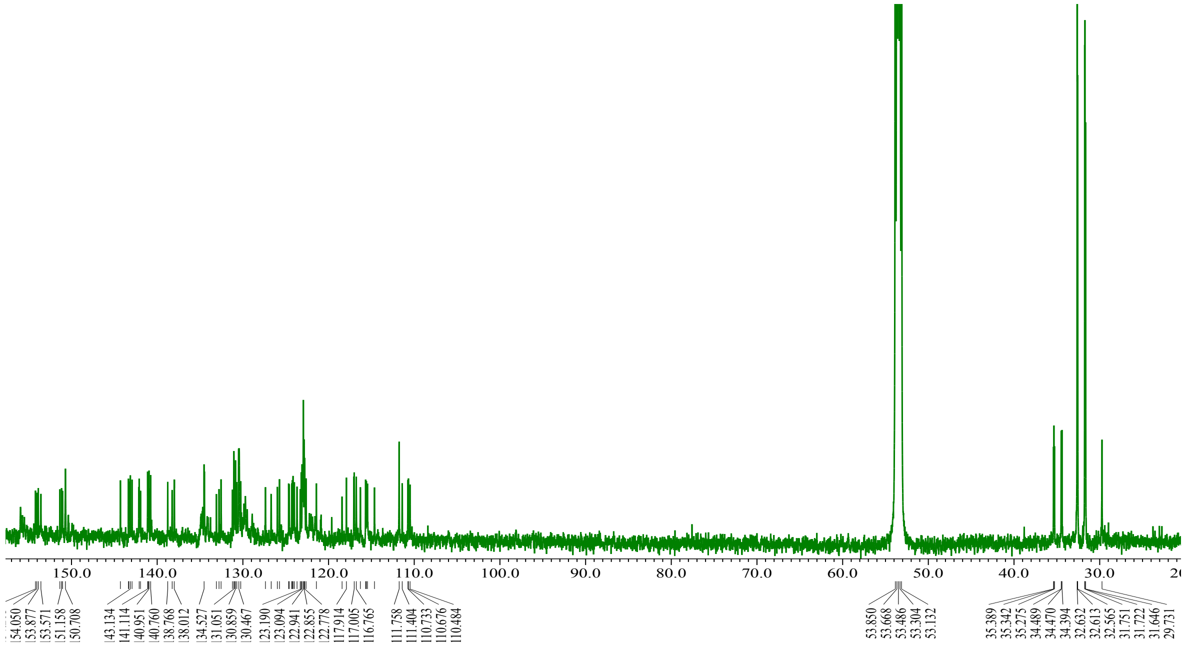

Figure S17. ^{13}C-NMR spectrum of **Ce^{IV}(Pc)(Pc3)** (CD_2Cl_2, 150MHz)

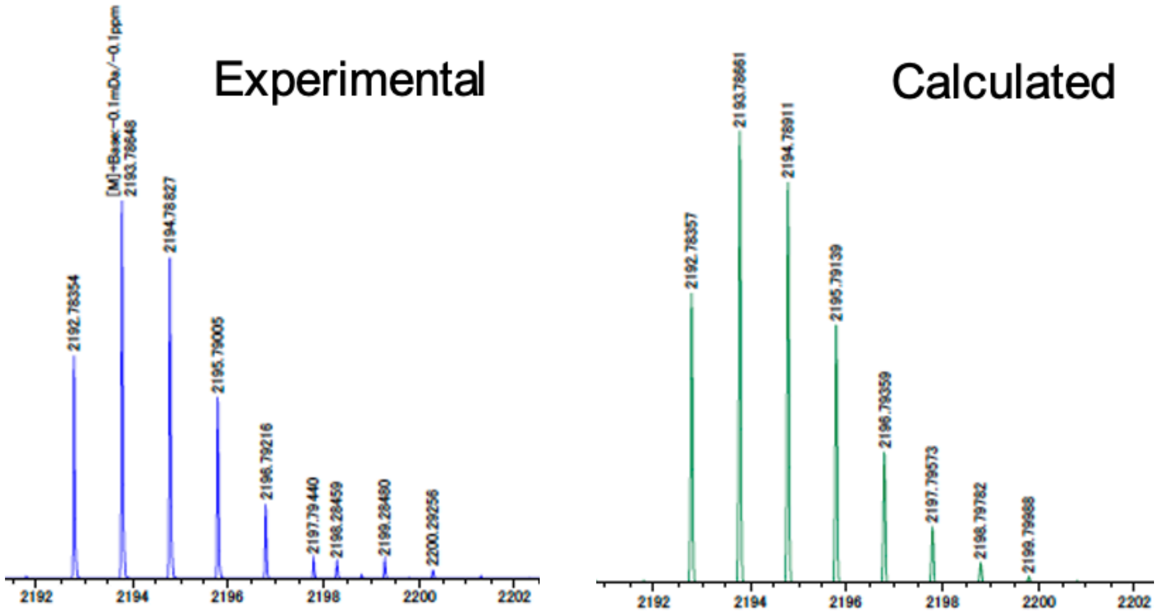

Figure S18. HR-MALDI-TOF MS for **Ce^{IV}(Pc)(Pc3).**

III. Crystallographic data (CCDC deposit number: 2324826)

III.1. Experimental details of single crystal X-ray analysis of complex **$Ce^{IV}(Pc2)_2$**

Single crystals suitable for the X-ray diffraction analysis were obtained by slow diffusion of MeOH into a $CHCl_3$ solution containing **$Ce^{IV}(Pc2)_2$**.

The single crystal X-ray diffraction data was collected on a Rigaku VariMax RAPID (1.2kW) diffractometer equipped with confocal mirror optics MoKα radiation and IP detector. The collected X-ray diffraction data were processed using the Rapid Auto software. The structure was solved by SHELXT and refined by full-matrix least-squares on *F*2 using the SHELXL-2019/2 and Olex2. The Solvent Mask function of Olex2 was employed to remove the disordered solvent molecules of electron density that could not be adequately modeled.

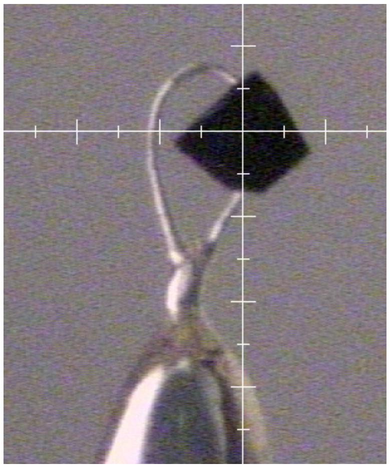

Figure S19. Picture of the crystal of **$Ce^{IV}(Pc2)_2$**.

III.2. Molecular structure of complex **$Ce^{IV}(Pc2)_2$**

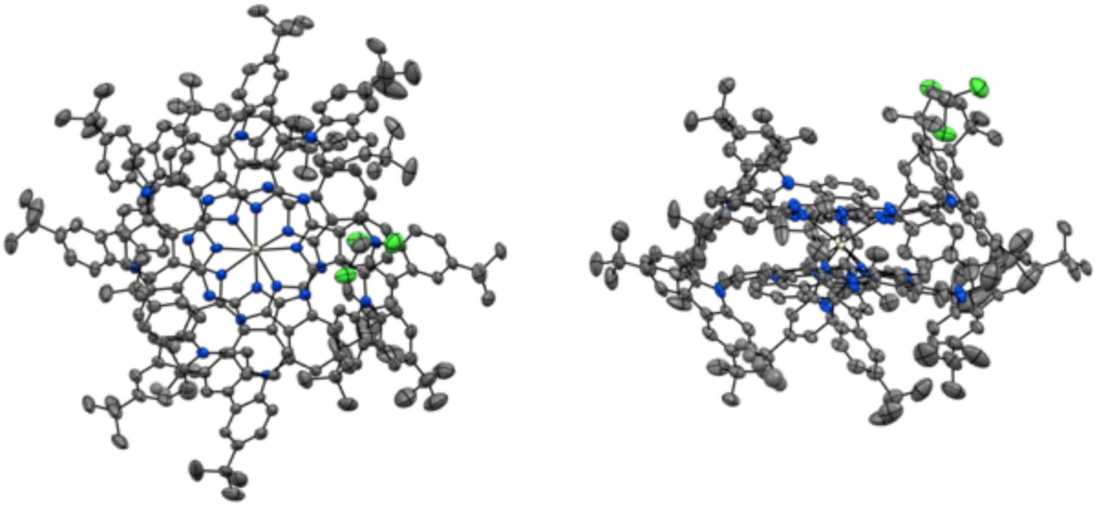

Figure S20. ORTEP representation of a top view (left) and a top view (right) of **$Ce^{IV}(Pc2)_2$**. Thermal ellipsoids are drawn at 50% probability. Hydrogen atoms are omitted for clarity.

III.3. Crystal data and structure refinement for complex **$Ce^{IV}(Pc2)_2$** (CCDC deposit number: 2324826)

Empirical formula	$C_{225}H_{217}CeCl_3N_{24}$
Formula weight	3503.68
Temperature	103.15 K
Wavelength	0.71075 Å
Crystal system	triclinic
Space group	P-1 (#2)
Unit cell dimensions	a = 20.5041(6) Å
	b = 23.9713(6) Å
	c = 24.9312(8) Å
	α = 64.455(5) °
	β = 71.217(5) °
	γ = 79.015(6) °
Volume	10448.1(7) $Å^3$
Z	2
Density (calculated)	1.114 g/cm^3
Absorption coefficient	0.313 mm^{-1}
F(000)	3688.0
Crystal size	0.14 x 0.12 x 0.04 mm^3
Theta range for data collection	1.741 to 25.351 °
Index ranges	-24<=h<=24, -28<=k<=28, -30<=l<=30
Reflections collected	145887
Independent reflections	38288 [R_{int} = 0.0720, R_{sigma} = 0.0621]
Completeness to theta = 25.351°	99.9 %
Absorption correction	Semi-empirical from equivalents
Max. and min. transmission	0.6225 and 1.0000
Refinement method	Full-matrix least-square on F^2
Data / restraints / parameters	38228 / 0 / 2327
Goodness-of-fit on F^2	1.031
Final R indices [I>2sigma(I)]	R1 = 0.0700, wR2 = 0.1793
R indices (all data)	R1 = 0.0958, wR2 = 0.1936
Largest diff. peak and hole	2.29 and -0.74 e.$Å^{-3}$
